# Insight into the interplay between mitochondria-regulated cell death and energetic metabolism in osteosarcoma

**DOI:** 10.3389/fcell.2022.948097

**Published:** 2022-08-22

**Authors:** Hong Toan Lai, Nataliia Naumova, Antonin Marchais, Nathalie Gaspar, Birgit Geoerger, Catherine Brenner

**Affiliations:** ^1^ CNRS, Institut Gustave Roussy, Aspects métaboliques et systémiques de l’oncogénèse pour de nouvelles approches thérapeutiques, Université Paris-Saclay, Villejuif, France; ^2^ INSERM U1015, Gustave Roussy Cancer Campus, Université Paris-Saclay, Villejuif, France; ^3^ Department of Pediatric and Adolescent Oncology, Gustave Roussy Cancer Campus, Villejuif, France

**Keywords:** osteosarcoma, mitochondria, regulated cell death, metabolic reprogramming, Metformin, ADI-PEG20

## Abstract

Osteosarcoma (OS) is a pediatric malignant bone tumor that predominantly affects adolescent and young adults. It has high risk for relapse and over the last four decades no improvement of prognosis was achieved. It is therefore crucial to identify new drug candidates for OS treatment to combat drug resistance, limit relapse, and stop metastatic spread. Two acquired hallmarks of cancer cells, mitochondria-related regulated cell death (RCD) and metabolism are intimately connected. Both have been shown to be dysregulated in OS, making them attractive targets for novel treatment. Promising OS treatment strategies focus on promoting RCD by targeting key molecular actors in metabolic reprogramming. The exact interplay in OS, however, has not been systematically analyzed. We therefore review these aspects by synthesizing current knowledge in apoptosis, ferroptosis, necroptosis, pyroptosis, and autophagy in OS. Additionally, we outline an overview of mitochondrial function and metabolic profiles in different preclinical OS models. Finally, we discuss the mechanism of action of two novel molecule combinations currently investigated in active clinical trials: metformin and the combination of ADI-PEG20, Docetaxel and Gemcitabine.

## 1 Introduction

Osteosarcoma (OS) is considered a rare disease and there has been no improvement in survival rate over the last 40 years. OS is the most frequent primary malignant bone tumor worldwide and predominantly affects children and young adults (Gill and Gorlick, 2021). For 80% of patients with OS, primary tumors arise in the metaphysis of long bones at extremities (distal femur, proximal tibia, proximal femur, and proximal humerus), which are the location of rapid bone growth (Isakoff et al., 2015) (Bielack et al., 2002). The disease demonstrates a high degree of malignancy and has a tendency to metastasize at early stages (Bielack et al., 2002). OS has two peaks of incidence: one between the ages of 5–24 years and the second peak from 60 to 85 years (Mirabello et al., 2009) (Whelan et al., 2012). Recent epidemiologic studies show that OS accounts 3%–5% of all pediatric cancers and 20%–40% of all bone tumors with an incidence rate of 2–4,2 cases/million per year (Rojas et al., 2021). Exemplary for other international trials, as part of the French OS2006/sarcoma-09 study, 376 patients underwent surgery after receiving intensive preoperative chemotherapy. 5-year survival rate outcomes were 1) 86% for localized disease and good histological response is; 2) 68% for localized disease and poor histological response; 3) 68% for metastatic disease and good histological response and 4) 24% for metastatic disease and poor histological response is (Gaspar et al., 2018) (Marchandet et al., 2021). Despite groundbreaking advances in other fields of pediatric oncology, therapeutic approaches for OS continue to demonstrate insufficient adequacy (Casali et al., 2018) (Mirabello et al., 2009). OS is considered as one of the most complex cancers and shows a high propensity to develop metastasis, mostly to the lung. This is one of the reasons for limited progress in treatment of this disease (Sheng et al., 2021).

To date, the main pillars of therapeutic management of OS are made of neoadjuvant chemotherapy with five major drugs: cisplatin (CIS), doxorubicin (DOX) and methotrexate (MTX), ifosfamide (IFOS) and etoposide (ETOP) (Gaspar et al., 2018), followed by surgical resection of the primary tumor along with the additional adjuvant chemotherapy after the surgery (Isakoff et al., 2015) (Edmonson et al., 1984) (Link et al., 1991) (Piperno-Neumann et al., 2016). Some patients acquire chemoresistance that impacts the survival, together with the development of metastasis (Whelan and Davis, 2018). Therefore, further studies are needed to understand OS tumorigenesis and progression, as well as for the development of OS-focused therapeutic and metastasis preventative approaches.

Across different cancer types, many basic and translational studies focused on the role of mitochondria in cancer cells, because these organelles are a central node in both, cellular metabolism, and cell fate decisions (Strasser and Vaux, 2020) (Carneiro and El-Deiry, 2020). Indeed, mitochondria are crucial for the maintenance of cellular homeostasis, energy production, regulating Ca^2+^ homeostasis and signaling, redox homeostasis, and cell survival or death decisions (Galluzzi et al., 2012) (Vasan et al., 2020). It is well established that mitochondria are the powerhouse of adenosine triphosphate (ATP) production *via* oxidative phosphorylation (OXPHOS) and the source of many biosynthetic intermediates (Mitchell, 1961). They function in a dynamic interconnected network with continuous communication to other cellular compartments such as the endoplasmic reticulum and the nucleus (Lai et al., 2022) (Galluzzi et al., 2012) (Green and Evan, 2002). Its functions are well described beyond the cellular level influencing whole organism physiology by regulating interactions between cells and tissues (Nunnari and Suomalainen, 2012).

Cancer metabolism is a flexible network of anabolic and catabolic reactions that allow cancer cells to respond to the cellular demands for rapid proliferation and adapt to harsh conditions of survival (Hanahan, 2022). Metabolic reprogramming influence many tumor stages, from transformation initiation to cancer progression (Faubert et al., 2020). To fulfill the energy requirements for cell growth and survival, including for the biosynthesis of proteins, lipids, and nucleotides, cancer cells must rewire their metabolism. The most famous phenomenon is the so called “Warburg effect,” classically used to describe the preference of use of cancer cells for glycolysis and lactate production under aerobic conditions (Warburg et al., 1927) (Liberti and Locasale, 2016) (Vander Heiden et al., 2009) (Schmidt et al., 2021). Besides enhanced glycolysis, there are other common metabolic adaptations, including prominent glutaminolysis to fuel the citric acid cycle and lipid biosynthesis (Cluntun et al., 2017). Increased pentose phosphate pathway activity aims to provide NADPH and pentose phosphate for nucleotide synthesis (Phan et al., 2014). Other metabolic pathways shown to contribute to tumorigenesis include fatty acid β-oxidation, folate-dependent serine and glycine metabolism, and the methionine cycle (Koundouros and Poulogiannis, 2020), (Rosenzweig et al., 2018). In line with these findings, metabolic reprogramming in cancer constitutes a decisive Achilles’ heel that attracts scientists and pharmacists to develop new classes of metabolic inhibitors to specifically target metabolic reprogramming. Understanding how metabolic reprogramming is acquired at different stages of tumor development and understanding its essentiality opens novel therapeutic opportunities by inducing cancer cell death in a personalized cancer concept (Stuani et al., 2019).

In this review, we provide a thorough overview of the current knowledge on the mitochondria-related RCD machinery in OS, focusing on the molecular mechanisms of apoptotic and non-apoptotic RCD. More specifically, we address apoptosis, ferroptosis, necroptosis, pyroptosis, and autophagy. Second, we will focus on the role of mitochondria and metabolic dysregulations in OS supported by fundamental findings by metabolomic and lipidomic analysis. We will finish by discussing on-going clinical trials of promising metabolism-related therapeutic targets showing the ability to induce cell death in OS.

## 2 Mitochondria-related regulated cell death in osteosarcoma

The Nomenclature Committee on Cell Death states that cell death pathways can be categorized into two distinct forms, accidental cell death and RCD. RCD includes both apoptotic and non-apoptotic cell death (ferroptosis, pyroptosis, necroptosis, etc.) (Galluzzi et al., 2018). Apoptosis is well-known to be the main form of RCD (Kerr et al., 1972). There are two principal apoptotic signaling pathways upon the activation of caspase-3 and caspase-8: cell death receptor (extrinsic) pathway and mitochondrial apoptotic (intrinsic) pathway (Carneiro and El-Deiry, 2020). It has been established that mitochondria are central initiators of the apoptotic intrinsic pathways, but they may also participate in other forms of RCD such as necroptosis, and ferroptosis (Nikoletopoulou et al., 2013).

### 2.1 Apoptosis

The intrinsic apoptosis pathway is triggered by various injurious stimuli such as oxidative stress, hypoxia, radiation, etc. (Elmore, 2007) (Danial and Korsmeyer, 2004). This type of programmed cell death requires the disruption of the outer mitochondrial membrane (OMM), known as mitochondrial outer membrane permeabilization (MOMP), allowing the release of pro-apoptotic proteins from the intermembrane space to the cytosol. However, since it is unclear how these apoptotic initiators cross the OMM and are thus released into the cytosol, several hypotheses have been proposed. These include opening of the PTP in response to over-production of ROS or Ca^2+^ overload (Biasutto et al., 2016), a large channel formed by Bax and/or Bak oligomers (Antignani and Youle, 2006) (Lovell et al., 2008), a channel formed by hetero-oligomers of VDAC1 and Bax (Banerjee and Ghosh, 2004) (Shimizu and Tsujimoto, 2000), or VDAC1 oligomers (Keinan et al., 2010) (Shoshan-Barmatz et al., 2013) (Keinan et al., 2013) (Weisthal et al., 2014) (Zalk et al., 2005). Despite the unclear mechanism, MOMP results in the release of cytochrome c (CytC) that, therefore, binds to and activates apoptotic protease activating factor 1 (Apaf-1) in cytosol. The heptameric quaternary structure of Apaf-1, thus, recruits cytoplasmic inactive pro-caspase-9 to form the apoptosome. Apoptosome is considered as the platform for the activation of pro-caspase-9, and other effectors such as caspase-3, -6, and-7 (Jiang and Wang, 2000, p. 1) (Bratton et al., 2001) (Chipuk et al., 2006). Moreover, MOMP induces the release of other apoptogenic factors that interact with other cellular organelles where they induce different pro-apoptotic functions. These apoptogenic factors include second mitochondria-derived activator of caspase (Smac)/direct inhibitor of apoptosis-binding protein with low pI (DIABLO), apoptosis-inducing factor (AIF), endonuclease G, and high-temperature requirement protein A2 (HtrA2/Omi) (Liu et al., 1996) (Verhagen et al., 2000) (Li et al., 2001) (Hegde et al., 2002). Importantly, once MOMP is induced and the transmembrane inner potential (ΔΨm) disrupted, a bioenergetic catastrophe occurs, manifesting by cessation of mitochondrial bioenergetic activities, ATP production arrest, ATP consumption, ROS production and pH changes (Kroemer et al., 2007).

**FIGURE 1:**
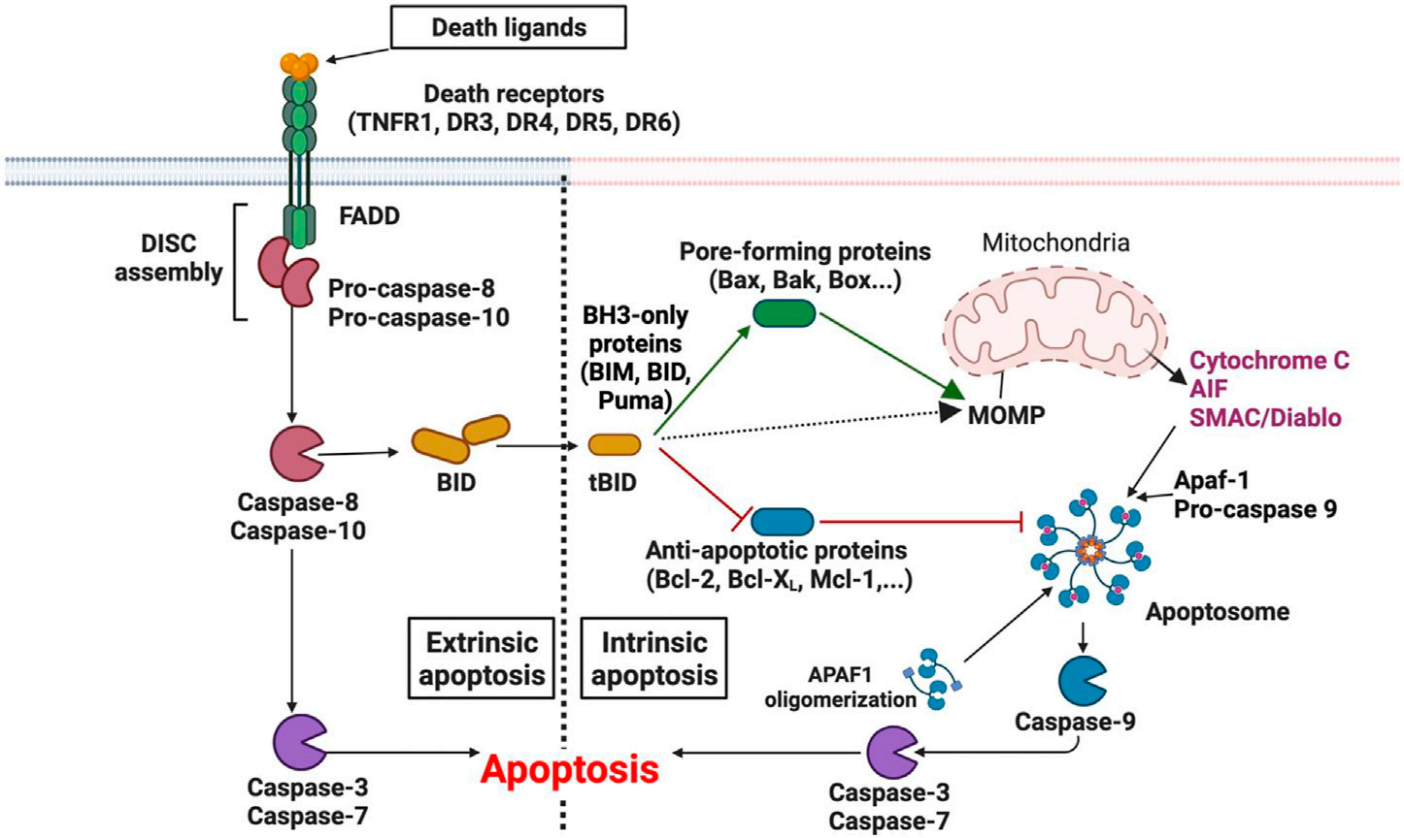
Molecular mechanisms of apoptosis.

The intrinsic apoptosis pathway is positively regulated by several pro-apoptotic members and negatively by anti-apoptotic proteins. Interestingly, for the past 30 years, a large set of proteins including both pro- and anti-apoptotic have been identified and categorized. These include proteins of the B-cell lymphoma 2 (Bcl-2) family with three subfamilies: anti-apoptotic, pore-forming proteins, and Bcl-2 homology 3 (BH3)-only proteins, whereby the classification is depending on the presence of the Bcl-2 homology domains. Anti-apoptotic members including, e.g., Bcl-2, B-cell lymphoma-extra-large (Bcl-X_L)_, B-cell lymphoma-w (Bcl-W), and myeloid cell leukemia 1 (Mcl-1) prevent the formation and activation of the apoptosome (Nijhawan et al., 2003) (Kharbanda et al., 1997) (Yang et al., 1997). Pore-forming proteins, in contrast, possess pro-apoptotic activities and include Bcl-2-associated X protein (Bax), Bcl-2 antagonist/killer (Bak) or Bcl-2 related ovarian killer (Box). The work by forming pores in the MOM, what is required for MOMP formation, as discussed below (Bleicken et al., 2014) (Borner and Andrews, 2014). The third group of proteins, namely BH3-only proteins, also has pro-apoptotic activities. Examples include Bcl-2-interacting mediator of cell death (BIM), BH3-interacting domain death agonist (BID), and p53-upregulated modulator of apoptosis (Puma). This family contains only one Bcl-2 homology domain that either activates pore-forming proteins and/or inhibits anti-apoptotic proteins to strictly regulate intrinsic apoptosis (Singh et al., 2019) (Zha et al., 1996, p. 2) (Figure 1).

Considered as an important apoptosis signal transduction pathway in cancer cells, extrinsic apoptosis pathway can be triggered by diverse DNA-damaging agents such as DOX, CIS, and ETOP (Müller et al., 1998) (Friesen et al., 1996). This type of apoptosis requires the binding of extracellular death ligands to death receptors. To date, there are six mammalian death receptors: tumor necrosis factor receptor 1 (TNFR1), Fas receptor, death receptor 3 (DR3), DR4 [tumor-necrosis-factor related apoptosis inducing ligand receptor (TRAILR1)], DR5 (TRAILR2), and DR6 (Nagata, 1997) (Nagata and Golstein, 1995) (Schneider et al., 1997) (Wiley et al., 1995) (Feinstein et al., 1995). This type of RCD requires generally three main steps: 1) binding of death ligands on death receptors; 2) formation of death-inducing signaling complex (DISC) by recruiting adaptor proteins including Fas-associated death domain-containing protein (FADD) and the initiator caspases. Cells can either promote the non-cytotoxic signaling-mediated cell survival *via* mitogen-activated protein kinases (MAPK) and/or nuclear factor-kappa B (NF-kB) surviving pathways (complex I) or cytotoxic signaling-mediated apoptosis (complex II) (Hsu et al., 1995) (Carneiro and El-Deiry, 2020). When NF-kB surviving pathway of complex I is activated, the cytotoxic complex II will be inhibited by caspase-8 inhibitor cellular FLICE-inhibitory protein (c-FLIP_L_) (Irmler et al., 1997). Complex II will only take place when the surviving pathway is insufficiently activated. Upon activating the cytotoxic signaling-mediated apoptosis axis, the assembly of the DISC complex stimulates the dimerization of two pro-caspase-8 molecules, results in self-cleavage and thus releases the active form of caspase-8 (Ozören and El-Deiry, 2002). Active caspase-8 then interact with the mitochondrial membrane and cleaves BID to become truncated BID (t-BID) by exposing its BH3-domain. This activates MOMP-mediated caspases to induce cell death (Schug et al., 2011) (Li et al., 1998) (Figure 1).

1) Extrinsic apoptosis: Binding of death ligands to their death receptors, induces the assembly of DISC which consists of adaptor proteins and pro-caspases (pro-caspase-8 and pro-caspase-10). Assembly of the DISC complex results in the activation of caspase-8 and caspase-10 and thus, caspase-3 and caspase-7. The crosstalk of extrinsic apoptosis and mitochondrial intrinsic apoptosis is mediated by the activation of caspase-8 and the cleavage of BID to tBID. tBID activates the MOMP-mediated caspases to induce cell death.
2) Intrinsic apoptosis can be activated by various intracellular stimuli that lead to the activation of pro-apoptotic proteins (including pore-forming proteins and BH3-only proteins). It induces the release of apoptogenic factors such as cytochrome *c,* AIF and SMAC/Diablo, followed by activation of the apoptosome including caspase-9, caspase-3, caspase-7 into the cytosol.

In OS, the dysregulated expressions of Bcl-2 family members have been observed and linked to patient prognosis, indicating that apoptosis could be a biomarker as well as an interesting therapeutic target. This was confirmed in a study of 49 high-grade OS patients, in which immunohistochemistry revealed that metastatic patients express higher levels of anti-apoptotic Bcl-2 in the primary tumor than non-metastatic patients. Moreover, low Bcl-2 expressing patients had a better prognosis than the high Bcl-2 expressing patients (TRIEB et al., 2013). Another study conducted by Wang et al. (2010) showed the protein and mRNA overexpression of anti-apoptotic Bcl-X_L_ in high metastatic osteosarcoma cell line (M8), in contrary to low metastatic osteosarcoma cell lines (Saos-2, MG63 and U2OS). Furthermore, high-expressing Bcl-X_L_ mRNA tissues from 72 OS patients without prior chemotherapy are correlated with lower prognosis. Interestingly, not only Bcl-X_L_ but other anti-apoptotic members (Bcl-2 and Mcl-1) also showed significantly higher protein expression in OS patient tissue samples, compared to non-tumor tissues samples. By knocking-down Bcl-X_L_, OS cell lines showed a significant reduction of cell proliferation and higher apoptotic rate, confirmed by the increase of caspase-3. *In vitro*, targeting apoptosis in OS has been shown to enhance chemosensitivity. Results supporting this observation include that genetically (siRNA) or pharmacologically (ABT-737, WEHI-539) inhibiting anti-apoptotic members such as Bcl-2 and Bcl-X_L_, in various OS cell line (MG-63, Saos-2, M8, U2OS, etc.) induced sensitivity to CIS and DOX treatment. In contrast, overexpression of pore-forming proteins such as Bax, induced chemosensitivity of the Saos2 OS cell line (Baranski et al., 2015) (Masuelli et al., 2020) (Eliseev et al., 2008) (Wang et al., 2010) (Baranski et al., 2015). Recently, drug combinations between Bcl-X_L_-inhibitor (A-1331852), Mcl-1 inhibitor (S63845) and/or Bcl-2 inhibitor (ABT-199/Venetoclax) were tested on a panel of pediatric cancer cell lines, including U2O2, MG-63 cell lines. Surprisingly, only dual inhibition of Bcl-X_L_ and Mcl-1 displayed a significant higher anti-tumoral effects in all tested pediatric cell lines and *in vivo* embryonic chicken model, rather than the combination with Bcl-2 inhibitor (Kehr et al., 2020). Despite the efficacy of those inhibitors in preclinical models, Venetoclax and dual inhibition of A-1331852/S63845 have been separately reported to cause acute liver toxicity in lung squamous cell carcinoma *in vivo* model and in clinical trial for hematological malignancies (NCT02265731), since these two proteins control the hepatic integrity in a gene-dose dependent fashion (Hikita et al., 2009) (Salem et al., 2019) (Weeden et al., 2018). In recent years, many new development of Mcl-1 inhibitors have been designed and entered clinical trial for different types of cancer as mono or combined treatment with Venetoclax (AstraZeneca, 2022) (AbbVie, 2021) (Institut de Recherches Internationales Servier, 2022). However, no clinical trial was launched, to our knowledge, to target those inhibitors in OS. Therefore, further studies are required to successfully target anti-apoptotic members to treat OS.

### 2.2 Non-apoptotic regulated cell death pathways

Non-apoptotic RCD processes are crucially involved in the induction of cancer development and progression, including OS (Tait et al., 2014). Although about 11 different RCD modalities have been described, mainly three have been associated with OS progression: ferroptosis, necroptosis, pyroptosis, and autophagy. Therefore, these RCD pathways can be considered as promising targets for OS treatment (Zhao et al., 2021) (Li et al., 2016). We will therefore limit this section to these three processes, excluding immunogenic cell death, among others, that are beyond the scope of this review.

#### 2.2.1 Ferroptosis

Ferroptosis is an important RCD process involved in various biological and pathophysiological processes (Dixon et al., 2012). Ferroptosis is driven by erastin-induced iron-dependent lipid peroxidation. Unlike apoptosis, ferroptosis is characterized by the rupture of the plasma membrane to expose inner components to the extracellular environment (Dolma et al., 2003) (Yang and Stockwell, 2008) (Yagoda et al., 2007).

**FIGURE 2:**
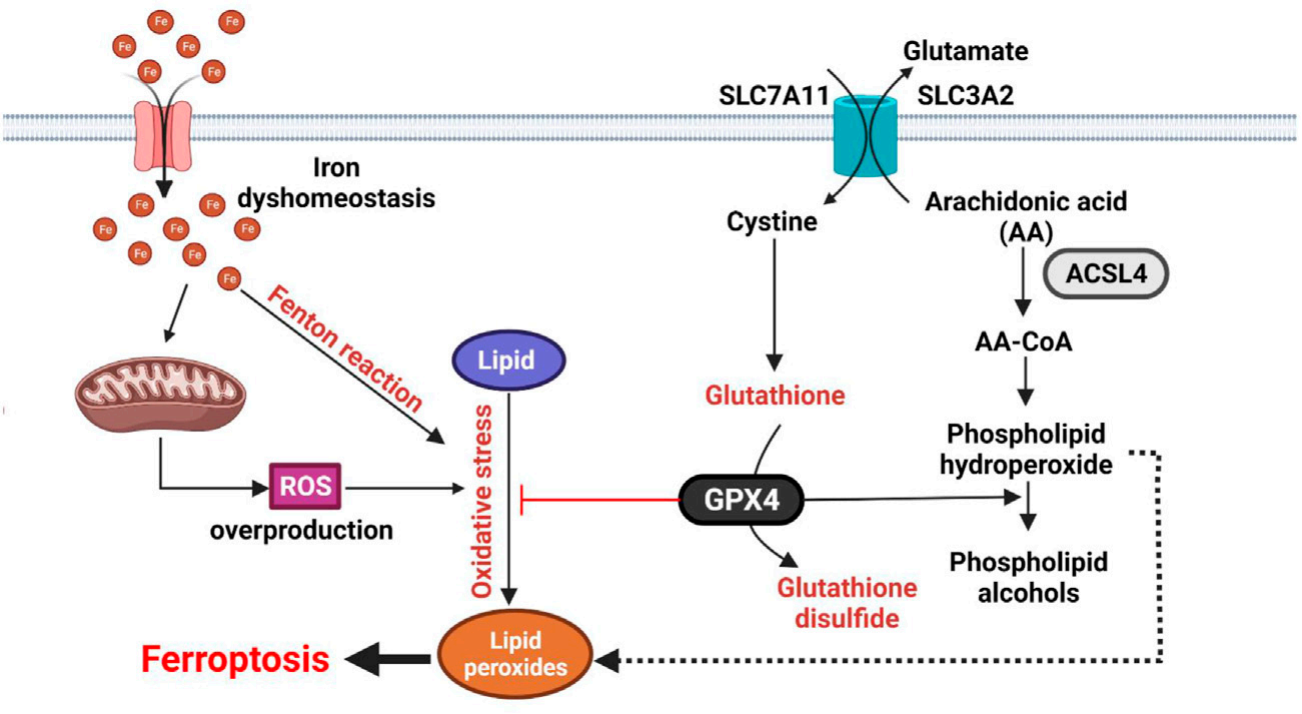
Molecular mechanisms of ferroptosis. Ferroptosis is characterized by oxidation of lipids upon inhibition of SLC7A11/SLC3A2 (Xc- system) transport complex or GPX4 activity. The reaction involved ROS and Fe^2+^. Ferroptosis is initiated by inhibition of the SLC7A11/SLC3A2 (Xc- system) transport complex or GPX4 activity. ROS and Fe^2+^ directly catalyze lipid peroxides to form damaging free radicals *via* the Fenton reaction. The SLC7A11/SLC3A2 antiporter is responsible for GSL biosynthesis by importing cystine into cytosol. GPX4 inhibits ferroptosis by transforming lipid peroxides into lipid alcohols.

The most important features of ferroptosis are the dys-homeostasis of lipid hydroperoxides and ferrous ion (Fe^2+^), which are induced by excessive reactive oxygen species (ROS), inducing oxidative stress (Galadari et al., 2017). As a guardian of redox balance, the glutathione (GSH) metabolic pathway participates in the regulation of ferroptosis (Kennedy et al., 2020). Glutathione peroxidase 4 (GPX4) is responsible for reducing the conversion from lipid peroxides into lipid alcohols in the presence of GSH and therefore acts as an important negative regulator of ferroptosis (Yang et al., 2014, p. 4). In this metabolic pathway, cystine is imported into the cytosol *via* the antiporter system (SLC7A11/SLC3A2), which is then converted into cysteine to produce GSH (Stockwell et al., 2017). Serving as a cofactor of GPX4, GSH is required for protecting the cells against oxidative stress and ferroptosis (Toyokuni et al., 2017). Depletion of either GPX4, SLC7A11, or GSH levels could lead to ROS overproduction and the accumulation of lipid peroxides (Friedmann Angeli et al., 2014) (Lachaier et al., 2014) (Eling et al., 2015). In the contrary increasing the abundance of intracellular cysteine, and GPX4 activity could lead to ferroptosis resistance. Beside the increased levels of Fe^2+^ and ROS, the increase of lipid oxidation products such as lipid hydroperoxides and subsequent reactive aldehydes [e.g., malondialdehyde (MDA) and 4-hydroxynonenal (4HNE)] are commonly used to evaluate ferroptosis (Shimada and Stockwell, 2016) (Lei et al., 2019) (Tang et al., 2021) (Figure 2).

Recent studies confirmed that ferroptosis can be induced by some natural anti-tumoral compounds [e.g., Bavachin and Phenethyl isothiocyanate (PEITC)] in *in vitro* and *in vivo* in OS models (Luo et al., 2021) (Lv et al., 2020). It was demonstrated that Bavachin treatment in OS cellular models (HOS and MG-63) significantly increased the intracellular ferrous ion, ROS, and MDA levels. Therefore, the ferroptosis regulators were found to be modulated in treated OS cell lines. In these experiments, it was also shown that Bavachin can inhibit SLC7A11, GPX4, phosphorylated signal transducer and activator of transcription 3 (p-STAT3). To explain the underlying molecular mechanisms, it was proposed that Bavachin can inhibit p-STAT3, subsequently stimulate tumor suppressor p53 and negatively regulate cysteine antiporter system (SLC7A11/SLC3A2) (Luo et al., 2021). Similar observations were found in PEITC-treated K7M2 OS cell line, showing the redox imbalance and the dysregulated Fe^2+^ metabolism (Lv et al., 2020). Interestingly, PEITC treatment also induces autophagy, which was confirmed by upregulation of autophagy markers such as Beclin1, conversion of LC3I to LC3II and p62. Hence, autophagy regulators such as mTOR and p-STAT3 were examined, demonstrating the inhibition of phosphorylated form of mTOR and STAT3. Given the important anti-tumoral effect of PEITC on OS cell lines, different doses of PEITC (from 30 to 90 mg/kg) were administrated in syngeneic orthotopic OS mouse model. Interestingly, tumor regression was significantly observed with 30 and 60 mg/kg doses, compared to the control group (Lv et al., 2020). All these results demonstrated that ferroptosis inducers could be proposed as a promising OS treatment strategy (Liang et al., 2019). However, ferroptosis inducers could be used only after detailed consideration and precise molecular screening, considering that ferroptosis-focused strategy may induce further complications in cancer patients. Indeed, it is worth noting that tumor-suppressor gene *TP53* can promote the accumulation of lipid peroxidation products, therefore promoting ferroptosis, and is broadly mutated in OS cell lines (Jiang et al., 2015).

Ferroptosis is closely related to autophagy and various interconnections between other forms of RCD have been demonstrated (Hou et al., 2016) (Gao et al., 2016). It is currently believed that the autophagy pathway can contribute to development of drug resistance, while the ferroptosis pathway can reverse this resistance (Zhou et al., 2019). To our knowledge, induction of ferroptosis in the treatment of OS are limited to chemotherapeutics, while alternative options, such as radiotherapy and immunotherapy, known to efficiently induce ferroptosis in other solid tumors, have not yet been reported to our knowledge (Zhao et al., 2021). Further studies are needed to evaluate the translational potential of inducing ferroptosis as a treatment strategy for OS.

#### 2.2.2 Necroptosis

**FIGURE 3:**
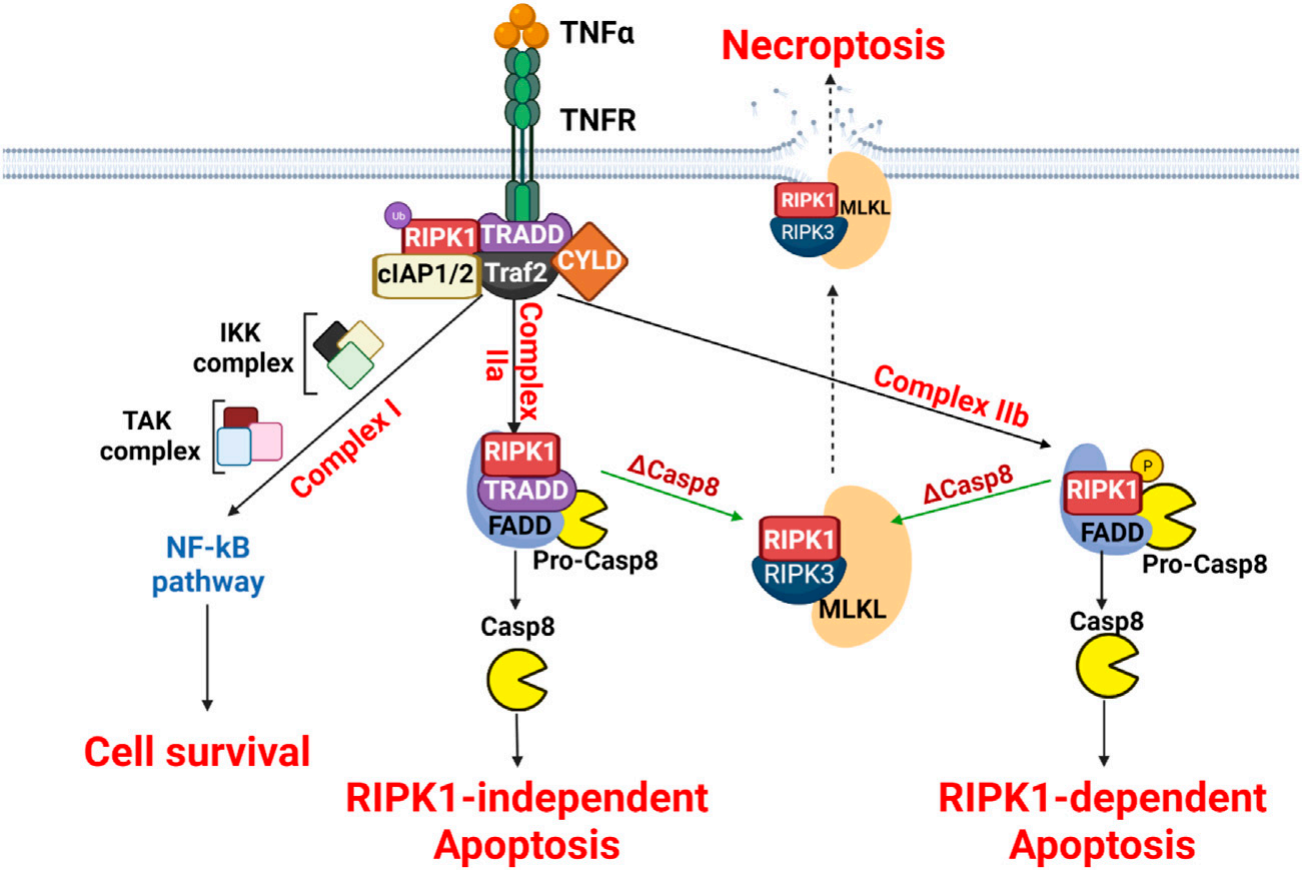
Molecular mechanisms of necroptosis. The scheme presents the three signaling pathways downstream of TNFR involving complex I, complex IIa and complex IIb formation. These complexes influence differentially cell fate. In the absence of caspase 8, necroptosis is then induced.

Necroptosis has previously been considered as the passive unprogrammed cell death, induced by external factors (toxins, various infections, physical traumatic influence, etc.) (Trump et al., 1997) (Jouan-Lanhouet et al., 2014). Based on current knowledge, however, it represents a form of RCD pathway and can be regulated similar to apoptosis (Christofferson and Yuan, 2010) (Robinson et al., 2019). Necroptosis and apoptosis share numerous molecular elements in the signaling transduction process, and could be modulated by the same effectors (Gupta et al., 2018) (Lin et al., 1999). Necroptosis is typically characterized by specific morphological characteristics such as swelling of the subcellular structures, permeabilization of the plasma membrane, mitochondrial depolarization, inflammation in the surrounding tissues (Khoury et al., 2020) (Figure 3). Understanding necroptosis signaling pathways could therefore contribute to the treatment of different pathologies, including cancers such as OS.

Necroptosis is initiated by the interaction between a wide range of stimuli such as tumor necrosis factor α (TNFα), interferon *γ* (IFγ) or lipopolysaccharide (LPS) and its receptors (Chen et al., 2019). The most studied prototype is the TNFα -/receptor TNFR pathway that has been thoroughly explored on the molecular level to induce necroptosis (Fulda, 2013). The binding of TNFα to its receptor TNFR results in the formation of the membrane signaling necroptotic complex I, containing tumor the necrosis factor receptor type 1-associated death domain protein (TRADD), FAS-associated death domain protein (FADD), RIPK1, cellular inhibitor of apoptosis protein 1/2 (cIAP1/2), and TNF receptor-associated factor 2 (TRAF2/5) (Han et al., 2011) (Vandenabeele et al., 2010). The stabilization of the complex is ensured by ubiquitination of RIPK1, that is mediated by cIAP1/2 and TRAF2/5. The ubiquitinated RIPK1 recruits IκB kinase (IKK) and TGFβ-activated kinase (TAK) complexes, leading to the activation of nuclear factor kappa B (NF-κB) pathway which promotes cell survival (Newton et al., 2014). In consequence, the formation of necroptotic complex I is considered an important checkpoint between cell survival and necroptosis (Oberst et al., 2011). To activate necroptosis, there are two crucial conditions 1) RIPK3 mandatory expression and 2) the inhibition of caspase-8 (Holler et al., 2000) (Vercammen et al., 1998). Following the inactivation of caspase-8, RIPK1 will be deubiquitinated by lysine 63 deubiquitinase (CYLD), thus forming the necroptotic complex II (O’Donnell et al., 2011)*.* Two different forms of necroptotic complex II are formed, (IIa and IIb), depending on the composition; complex IIa contains TRADD, RIPK1, FADD, and caspase-8 while complex IIb only contains RIPK1, FADD, and caspase-8 (Amin et al., 2018). For the complex IIa, the presence of caspase-8 induces RIPK1-independent apoptosis while for the complex IIb, without TRADD, phosphorylated RIPK1 activates caspase-8, resulting in RIPK1-dependent apoptosis (Micheau and Tschopp, 2003). Interesting, only when caspase-8 is inhibited the necrosome forms with the presence of RIPK1, RIPK3, and MLKL, thus promoting necroptosis-associated pore formation (Pasparakis and Vandenabeele, 2015).

Upon TNFα binding to TNFR, complex I is formed by recruiting adaptor proteins (RIPK1, Traf2, cIAP1/2, and CYLD) using TRADD as a platform. The ubiquitination of RIPK1 results in the recruitment of the IKK complex and/or TAK complex, promoting the NF-κB survival pathway. The complex II activation requires the deubiquitylation of RIPK1, thus forming the complex IIa (containing RIPK1, TRADD, and FADD) and pro-caspase-8 or the complex IIb (containing RIPK1, FADD, and pro-caspase-8). Both complex IIa and IIb can induce apoptosis. Therefore, activation of necroptosis is obtained only when caspase-8 is inhibited or depleted by recruiting RIPK1, RIPK3, and MLKL to form the necrosome. MLKL-mediated necroptosome causes the disruption of plasma membrane and cell lysis.

Necroptosis can be considered as a mitochondrial-mediated RCD, because the receptor-interacting protein kinase-1/-2/pseudokinase mixed lineage kinase domain-like (RIPK1/3/MLKL) necrosome complex induces mitochondrial dysfunction and thereby necroptosis (Wang et al., 2012) (Marshall and Baines, 2014) (Tian et al., 2020). The RIPK1/3/MLKL necrosome complex depends on mitochondrial functioning and can enhance oxidative respiration, ROS generation, translocation of Bax/Bak to mitochondria, and various metabolic enzymes in the mitochondrial matrix (Van Herreweghe et al., 2010) (Gan et al., 2019) (Tischner et al., 2012). Targeting necrosome complex could offer effective therapeutic strategies for OS treatment, according to the existing *in vitro* and *in vivo* studies (Sun et al., 2015) (Fu et al., 2013). For example, Shikonin, a natural compound, extracted from Chinese medicinal herbs, was demonstrated to induce necroptosis in both primary and metastatic OS by upregulating RIPK1 and RIPK2 (Fu et al., 2013).

However, necroptosis involvement in OS progression is still ill-defined and open new opportunities for researchers to deeply investigate.

#### 2.2.3 Pyroptosis

Pyroptosis is a regulated cell death type, which was discovered relatively recently and was characterized as morphologically different from apoptosis, ferroptosis and necrosis (Ding et al., 2016) (Shi et al., 2017). Pyroptosis is considered as the inflammatory type of regulated cell death that requires membrane pore-forming proteins and is associated with the morphological changes of cells, including swelling, with cell lysis and release of various proinflammatory factors (Xia et al., 2019). It is believed that pyroptosis could generate a tumor-promoting milieu due to the circulation of numerous inflammatory factors. In parallel, pyroptosis could prevent tumor growth by inducing a regulated cancer cell death (Xia et al., 2019) (Wu et al., 2021).

Pyroptosis depends on the formation of plasma membrane pores by the pore-forming proteins, in particular, gasdermin protein family (Galluzzi et al., 2018). Gasdermin D (GSDMD) as a key member of the gasdermin protein family is a primary substrate for the caspase family and after cleavage by activated caspases, the N-terminal fragment of GSDMD (N-GSDMD) could be oligomerized, leading to the pores formation and then, pyroptosis initiation (Lu et al., 2021). It is shown that genes linked to the pyroptosis are associated with the proliferation and invasion of tumor cells in different cancer types, strongly indicating the cancer prognosis (Lin et al., 2020) (Ye et al., 2021) (Tang et al., 2020) (Wang et al., 2018). It is obvious that pyroptosis-associated genes are involved in the osteosarcoma progression, however, the precise molecular mechanisms are still needed to be clarified.

**FIGURE 4:**
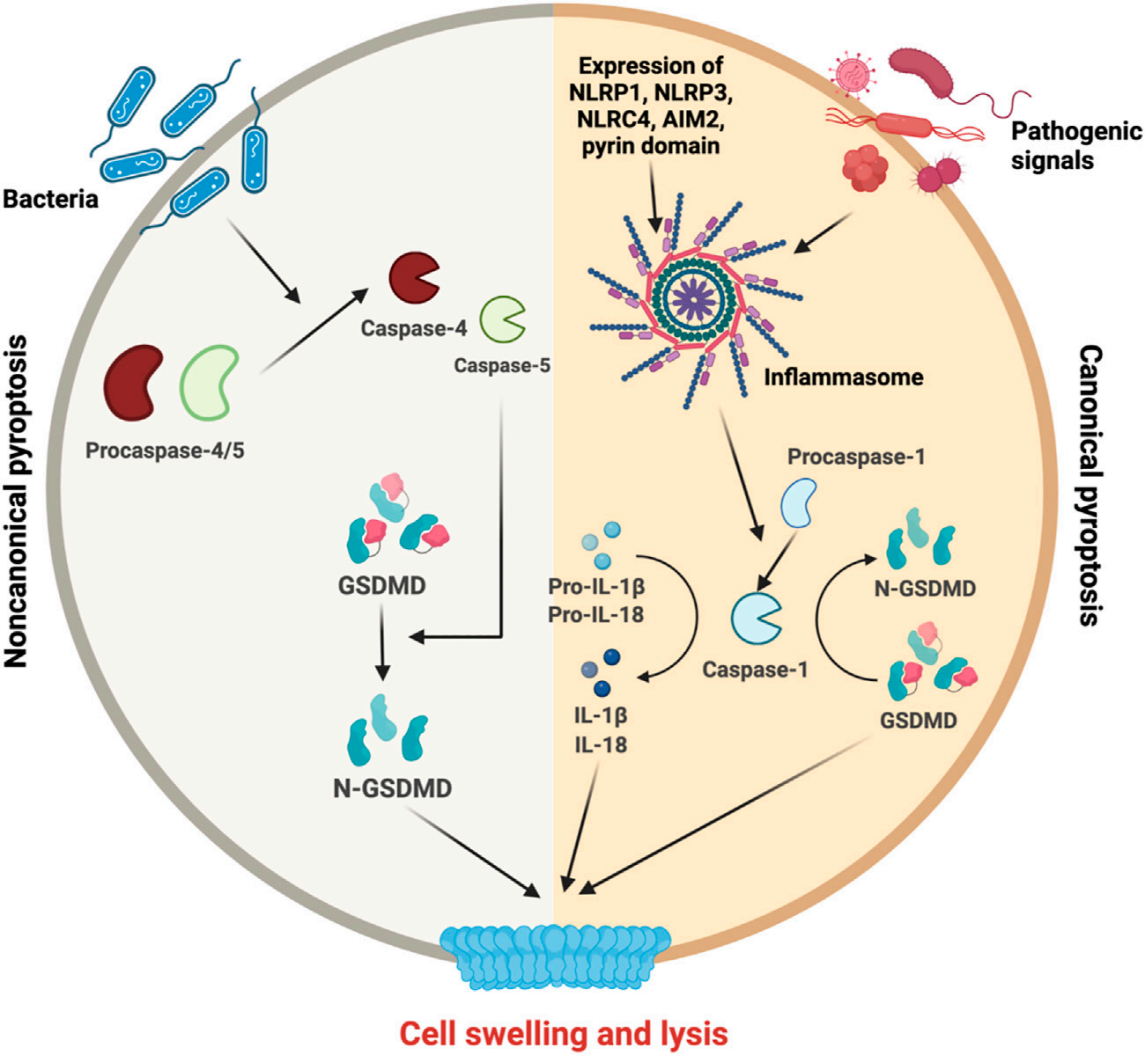
Molecular mechanisms of pyroptosis. Upon the initiation of noncanonical pyroptosis or canonical pyroptosis, different inflammatory actors are triggered. On the left side, pro-caspase 4/5 are cleaved for their maturation and participate in noncanocical pathway to cleave GSDMD into its active form N-GSDMD. On the right side, canonical pathway requires inflammasome formation to activate the caspase-1. The caspase-1 is necessary for GSDMD cleavage and IL-1β and IL-18 maturation, release and final steps of cell swelling and lysis.

Canonical inflammasome-induced pyroptosis can be induced by various pathogenic signals and is associated with the formation of inflammasome complex. Several inflammasomes (NLRP1, NLRP3, NLRC4, AIM2, and pyrin domain) were identified to interact with caspase activation and recruitment domain (PYD/CARD) (Shi et al., 2017) (Wu et al., 2021). Inflammasomes interact with the adaptor protein apoptosis associated speck like proteins (ASC) and pro-caspase-1 (He et al., 2016). Activation of pro-caspase-1 is needed for the cleavage of GSDMD and N-GSDMD production (Shi et al., 2015) (Van Opdenbosch and Lamkanfi, 2019). In addition, active Caspase-1 could cleave and activate pro-IL-1β and pro-IL-18 allowing their release *via* the membrane pores (Lacey et al., 2018) (Figure 4).

In contrast, noncanonical inflammasome-induced pyroptosis could be triggered by lipopolysaccharide (LPS) and bacteria and is independent of the inflammasome pathway. It is associated with the activation of caspase-4/5 *via* their oligomerization. Finally, GSDMD is cleaved in N-GSDMD leading to cell swelling and lysis (Yang et al., 2015) (Shi et al., 2014) (Fang et al., 2020).

Recently, even is the number of patients investigated is limited, differential expression analysis of the microarray data from the TARGET and GTEx databases identified 46 differentially expressed pyroptosis-related genes in osteosarcoma (Zhang et al., 2021). This evaluation of the association between the pyroptosis-related signature scores and clinical characteristics with immune microenvironment in osteosarcoma indicates that the pyroptosis-associated prognostic signature could be crucial for the osteosarcoma diagnosis and prognosis (Zhang et al., 2021).

To date, the role of mitochondria in OS-induced pyroptosis is still unknown. As it has been shown that the Ragulator-Rag-mTORC1 pathway and mitochondrial ROS are required for pyroptosis in macrophages, it could be important in the future to examine more deeply how mitochondria could affect this cell death modality in OS (Evavold et al., 2021).

#### 2.2.4 Autophagy

**FIGURE 5:**
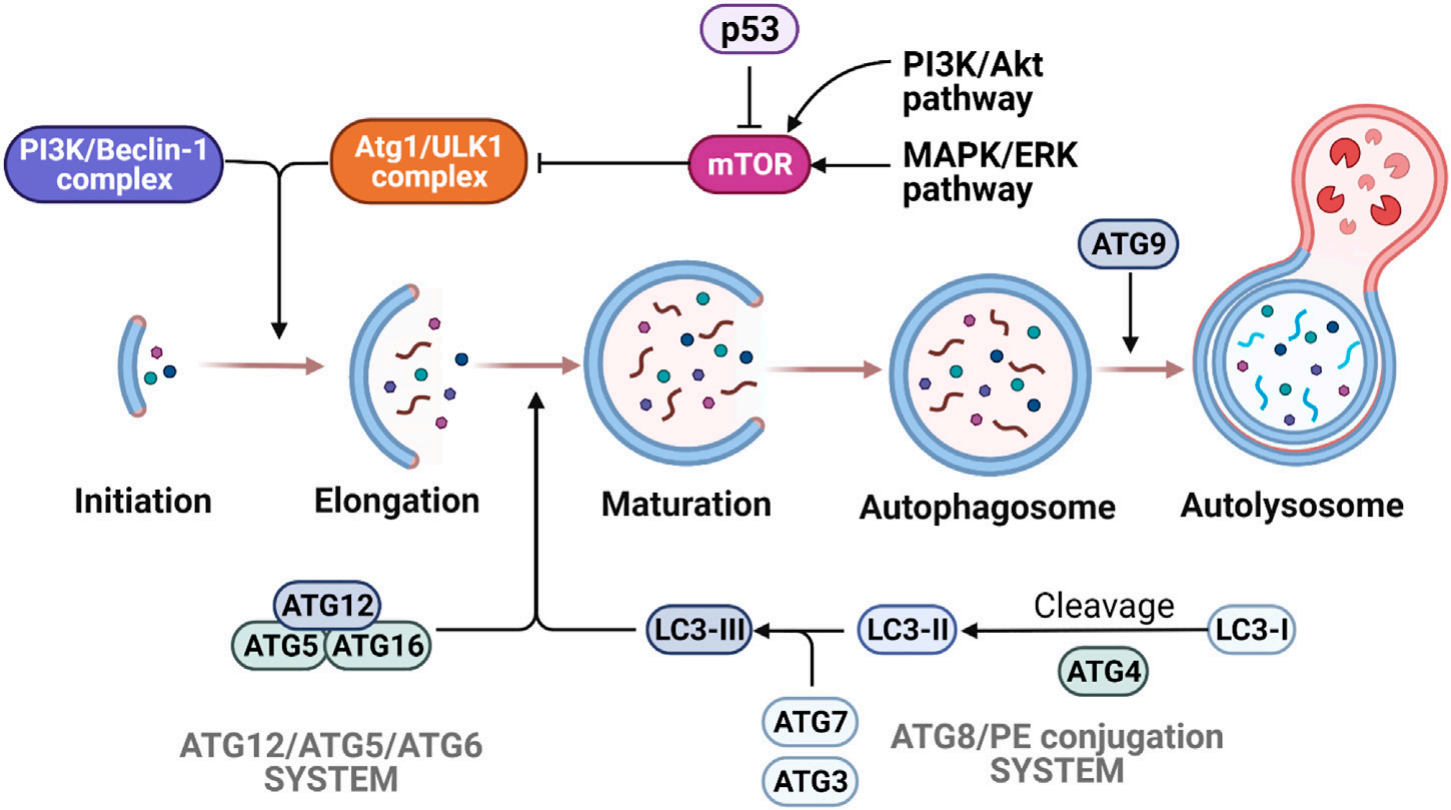
Molecular mechanisms of autophagy. The various steps of autophagy process are depicted following mTORC1 regulation by p53, AMPK and MAPK/ERK signaling. Autophagy is activated by the initiation complex ULK1/Atg1 and PIK3/Beclin-1 complex, which enables the autophagosome formation. The elongation and maturation depend on two ubiquitin-like conjugation systems (Atg12/Atg5/Atg6 system and Atg8/PE system). Many signaling pathways are involved in the regulation of autophagy. Due to the activation of p53 signaling or AMPK, activity of the mTORC1 complex is decreased and triggers autophagy activation. On the other hand, the activation of PI3K/Akt and MAPK/Erk pathways induce the increase of mTORC1 activity that results in the inhibition of autophagy.

Autophagy is a catabolic pathway that is activated by numerous factors including cellular stress such as nutrient deprivation, hypoxia or ROS overproduction (Levine and Kroemer, 2008). This process is characterized by the degradation of cytoplasmic components and is regulated in defined sequential steps: 1) induction of autophagy by cellular stress; 2) phagophore formation for damaged organelles; 3) autophagosome formation by elongating and closing the phagophore; 4) autolysosome formation by fusing the autophagosome with the lysosome to degrade the damaged organelles (Glick et al., 2010) (Figure 5). For over 30 years, a large set of autophagy-related genes (Atg) has been discovered and categorized into five groups according to their functions: 1) Atg1/UNC-51-like kinase (ULK-1) complex to initiate autophagy, 2) class III phosphatidylinositol three kinase (PI3K)/Beclin-1 complex to form the autophagosome, 3) the Atg12/Atg5/Atg6 conjugation system for promoting the formation of autophagy precursors by elongating the vesicle, 4) the Atg8 (LC3)/phosphatidylethanolamine (PE) conjugation system to form a tight membrane-associated form, and 5) Atg9 for the autophagosomal membrane expansion (Nakatogawa et al., 2009) (Matoba et al., 2020). In the absence of cell stress, the mammalian target of rapamycin (mTOR) signaling pathway negatively inhibits ULK1, thus preventing autophagy. Upon cellular stress, mTOR is inhibited, releasing ULK1 triggering autophagic activity (Díaz-Troya et al., 2008) (Figure 5). In cancer cells, the overactivation of oncogenic mTOR signaling pathway is frequently observed and mTOR inhibitors have been actively developed in recent years (Hua et al., 2019). In OS, the activation of mTOR pathways is associated with tumor growth and proliferation, metastasis, inhibition of apoptosis and suppression of autophagy, hence becoming an attractive therapeutic target (Kuijjer et al., 2014) (Houghton et al., 2012) (Gordon et al., 2008) (Mu et al., 2013) (Wang et al., 2014) (Song et al., 2015). While mTOR inhibitor has a limited efficacy in cancer cells, the combination of mTOR inhibitors and other drugs demonstrated synergistic effects. Different combinations were explored in the clinical trials. For example, Cixutumumab [humanized IgG1-monoclonal-antibody-targeting insulin-like growth factor-1 (IGF-1R)] combined with Temsirolimus (mTOR inhibitor) showed no robust differences in median progression-free survival end point for both IGF-1R positive patients and IGF-1R negative patients (Schwartz et al., 2013). Another clinical study for unresectable high-grade OS after standard treatment with Sorafenib (multikinase inhibitor) and Everolimus (mTOR inhibitor) also failed to demonstrate 50% of 6-month progression free survival endpoint as previously designed in the study (Grignani et al., 2015). Although several pre-clinical data suggested that mTOR inhibitors showed anti-tumoral effects in OS models, more clinical trials are nonetheless needed to confirm this attractive target.

According to many studies, autophagy is known to play a dual role as a promoter of cell survival and chemosensitivity. In normal conditions, autophagy prevents organelle dysfunction and maintains normal cellular biosynthesis. Chemo-resistant OS cells exhibit a high autophagy rate, especially CIS-resistant cell lines (Jiang et al., 2017) (Mukherjee et al., 2017). Through autophagy induction, they also observed the stimulation of many resistance-mediated factors such as high mobility group box 1 protein (HMGB1), Heat Shock Protein 90 Alpha Family Class A Member 1 (HSP90AA1), and Glial cell-derived neurotrophic factor family receptor alpha 1 protein (GFRA1). Moreover, knock-down of Beclin-1, an essential actor for autophagosome formation displays reduced cell invasion and metastasis rates. These results were confirmed *in vivo* in OS mouse xenograft models resulting in reduced tumor growth. Beclin-1 knocked-down cell lines show significantly increased sensitivity to classic anticancer agents used to treat OS, such as DOX, CIS, and MTX as proven in HOS, MG63, and U2OS cell lines (Zhang et al., 2015). Similarly, Atg14 knock-down Saos2 cell lines rendered more sensitive to CIS (Zhao et al., 2014), suggesting that the high autophagic rate can strongly contribute to OS chemoresistance. Thus, many autophagy inhibitors are recently under investigation in combination with conventional drugs such as CIS and DOX to sensitize OS cell death (Sui et al., 2013).

While the protective role of autophagy is well known to provide many advantages for OS development, the role of autophagy to induce cell death is still under debate. Increasing evidence suggests that autophagy also has anti-tumoral roles under certain conditions. Through mechanisms, yet to be confirmed, autophagy also is able to promote apoptosis (Thorburn, 2008). Many drugs stimulate both autophagy and apoptosis but pharmacological inhibition of autophagy results in a decrease of apoptotic markers, such as caspase-3 and poly (ADP-ribose) polymerase (PARP) cleavage, showing the effect of autophagy-induced apoptosis (Wang et al., 2013) (Li et al., 2015). Recently, by analyzing 394 tumor specimens from 260 OS patients in various stages of OS development, Livingston et al. (2018), suggested that positivity for puncta of the autophagy marker LC3B + can be used as prognostic markers for better survival after neoadjuvant chemotherapy. They therefore highlighted the beneficial role of autophagy in this context.

### 2.3 Interplay between mitochondrial-related cell deaths and metabolism in osteosarcoma

In many cancers, the interconnections between metabolism and cell death are mainly mediated by mitochondrial dysfunction 1) pro-apoptotic MOMP, 2) ROS- or Ca^2+^-dependent mitochondrial permeability transition pore (mPTP), 3) ATP production by OXPHOS and glycolysis, 4) synthesis of certain biosynthetic intermediates (Green et al., 2014), opening the perspective to identify new factors/checkpoints connecting these processes. Unfortunately, knowledge is limited in OS in comparison to other cancer types and deserves more research efforts. Thus, the search for connection between cell death and metabolism might be stimulated by the bioinformatic analysis of genomic and genetic pediatric OS patients’ data (Berlanga et al., 2022).

Glucose and glutamine are important fuel sources for cancer cells (DeNicola and Cantley, 2015). Whereas this is almost exclusive in cell culture, also *in vivo* they remain important contributors across a wide variety of cancer types (Hui et al., 2017). In metabolic stress conditions, especially nutrient starvation, cells undergo cell cycle arrest and MOMP-driven apoptosis. For example, glucose deprivation first reduces ATP production rate and increases the AMP/ATP ratio, inducing the activation of AMP-activated protein kinase (AMPK) to arrest the cell cycle (Kim et al., 2010). In parallel, AMPK activation also causes the phosphorylation of p53 to induce p53-dependent apoptosis (Okoshi et al., 2008). Together with AMPK, p53 increases levels of pro-apoptotic BH3-only proteins (BIM and Puma) and pore-forming protein (Bax) to sensitize cells to mitochondria-mediated apoptosis (Zhao et al., 2008) (Chi et al., 2000). Similarly, glutamine deprivation induces both extrinsic and intrinsic apoptotic pathways but these events are cell type-specific (Fuchs and Bode, 2006). This can stimulate the activation of different caspases either -2, -3, -8, and/or -9, cleaved-PARP expression, translocating pro-apoptotic protein Bax or releasing apoptogenic factor CytC, depending on the cell line (Paquette et al., 2005) (Fuchs et al., 2004).

The mitochondrial permeability transition (mPT) is a pathophysiological state of the mitochondrial inner membrane (MIM). It is related to specific permeability conditions, including Ca^2+^ overload, oxidative stress, increased phosphate concentrations, decreased ATP availability or various stress conditions (Zoratti and Szabò, 1995) (Baumgartner et al., 2009) (Seidlmayer et al., 2015) (Crompton, 1999). The mPT is mediated by the multi-protein pore between MIM and MOM called the mPTP. Opening of mPTP results in loss of ΔΨm and ATP synthesis interruption, being directly regulated by the concentration of mitochondrial Ca^2+^ (mtCa^2+^) levels (Marzo et al., 1998) (Bernardi et al., 2015) (Duchen, 2000). The functional dualism of mtCa^2+^ is an important factor of mPTP induction: physiological levels of mtCa^2+^ could activate transient opening of mPTP, while mtCa^2+^ overload leads to significant pathological changes, resulting in sustained mPTP opening. This subsequently induces mitochondrial dysfunction and cell death (Morciano et al., 2021). Of note, modulation of mtCa^2+^ caused by the dysfunctions of Ca^2+^ transporters or oncogenic signaling pathways can regulate the opening of mPTP activity. Moreover, mPTP opening is strictly regulated by series of regulators, including voltage-dependent anion channel 1 (VDAC1) on the MOM, adenine nucleotide translocator (ANT) on the MIM, Hexokinase II (HKII), glycogen synthase kinase 3 *β* (GSK3β), cyclophilin D (CypD) and also members of apoptotic Bcl-2 family (Baines et al., 2005) (Kroemer et al., 2007) (Bonora et al., 2022).

In solid tumors, including OS, cancer cells avoid the opening of the mPTP, however, the precise molecular mechanisms have not been identified. One possible mechanism is related to the Warburg effect with an increase in aerobic glycolytic ATP production (Warburg et al., 1927). The oxygen independent continuous ATP production through glycolysis avoids ATP depletion and at the same time promotes lactate accumulation. This secondary product of the Warburg effect, lowers the extracellular pH levels, thus inhibiting mPTP activation and contributing to evade cell death (Bonora and Pinton, 2014). Hypoxia-inducible factor 1-alpha (HIF1α) is another potential mechanism to explain the mPTP-mediated cell death avoidance. HIF1α acts as a transcription factor that stimulates the expression of glycolysis-related genes, including HKII and therefore promoting the Warburg effect (Gogvadze et al., 2008). Recent studies reported that HIF1α-mediated HKII over-expression, results in mPTP inhibition (Gwak et al., 2005). Therefore, HKII becomes an attractive target for cancer therapy. HKII can interact with other regulators of the mPTP, namely VDAC1, to facilitate VDAC interaction with the ANT to form a contact site between MOM and MIM facilitating thereby the exchange of cytosolic ADP for mitochondrial ATP. Subsequently, ATP from mitochondria bind to active sites on HKII, resulting in activation of HKII for glycolysis (Azoulay-Zohar et al., 2004) (Beutner et al., 1996). Moreover, VDAC1 is an important effector due to its role in transporting metabolites, Ca^2+^, anions, cations, etc.; across the MOM and is also known for its interaction with anti-apoptotic proteins (Bcl-2 or Bcl-X_L_) (Shoshan-Barmatz et al., 2017). Targeting HKII and/or VDAC1 by chemical agents, genetic manipulation, microRNA or peptide-based therapies have been demonstrated induce significant inhibition of glycolysis, loss of ΔΨm, and induce cell death (Galluzzi et al., 2008) (Li et al., 2017, p. 2) (Sun and Zhang, 2017, p. 2) (DeWaal et al., 2018).

Immunohistochemical analysis of 30 OS patient derived specimens showed the overexpression of HKII in 83.3% OS specimen, compared with normal bone tissues. This overexpression has been also found in a panel of OS cell lines (MG63, HOS, Saos2, and U2OS). Therefore, knock-down of siRNA in MG63 and U2OS led to decrease Warburg effect and remarkably increases apoptotic rates. However, 2-Deoxy-D-Glucose (2-DG)—a glycolysis inhibitor through HKII did not display any significant changes in cell viability. The authors suggested that the current inhibitors such as 2-DG or 3-bromopyruvate are not efficient due to their low specificity. Subsequently, targeting the interaction of HKII and VDAC could be beneficial to reduce Warburg effect observed in OS (Zhuo et al., 2015). Beside those chemical inhibitors, the research of miRNA–endogenous RNA has been elucidated in many human cancers and showed the potential clinical application, including OS. Recently, two miRNAs, namely miR-615 and miR-185 were discovered to target HKII and reduce OS cell viability and metastasis. MiR615 was found to be significantly downregulated in 92 OS patient tissues and correlated with poor clinical outcomes. Mimicking miR-615 in HOS cell line reduce cell proliferation and metastatic rates by directly targeting HKII while inhibiting miR-615 showed the opposite effects. Therefore, epithelial-mesenchymal transition (EMT) and PI3K/Akt pathway were found to be dysregulated in miR-615 mimicking HOS cell line (Sun et al., 2020, p. 2). In other study, total of 30 OS tissues were compared with adjacent normal tissues, showing the downregulation of miR-185 in OS tissues. This observation was also confirmed in OS cell lines (MG-36, U2OSS, HOS, Saos2), compared with human osteoplastic cell line (NHOst). MiR-185 is also able to directly target HKII and reduce glycolytic profile and cell viability of OS cell lines (C. Liu et al., 2019a, p. 2). Taken together, these two miRNAs could become novel therapeutic candidates for OS treatment in the future. However, there are still many challenges of miRNA application in the future to overcome, such as, technical development and unclear understanding of biological characteristics of miRNAs (Cui et al., 2019). Taken together, understanding the interplay between metabolism and cell death could be useful to identify critical cell fate checkpoint and help the development of anticancer metabolic inhibitors.

## 3 Mitochondria and metabolic reprogramming

### 3.1 The dysregulation of energy metabolism and substrate energy use

Cancer cell lines have been instrumental models to discover tumor proliferation mechanisms, characterize functional profiles, and as models for drug discovery over sever decades (Masters, 2002). Unfortunately, the number of established OS cell lines is relatively low, compared to other cancer types (Mohseny et al., 2011). Therefore, the knowledge of heterogeneity in metabolic adaptation of OS is limited.

Recently, the metabolic comparison between the noncancerous osteoblast cell line (hFOB), parental OS cell lines (Saos2 and HOS), and its metastatic subtypes (LM7 and 143B), has helped deepen our understanding (Table 1). By examining the morphology of mitochondria, hFOB, Saos2, and HOS cell lines display elongated mitochondria. Bioenergetic values such as OCR and ECAR were not significantly different between hFOB, Saos2, and HOS with a partial decrease of ΔΨm and impaired mitochondrial integrity, thus hinting towards involvement of mitochondrial dysfunction in OS oncogenesis. Furthermore, apoptotic parameters of Saos2 and HOS were examined, showing significantly decreased cytosolic CytC protein expression in the HOS and Saos2 cell lines, compared with hFOB, following the increase of anti-apoptotic Bcl-2 protein expression while caspase-3 remained unchanged (Giang et al., 2013).

**TABLE 1:** Characteristics of dysregulated energy metabolism and substrate energy use in OS models.

Non-metastatic model		Metastatic model		References
Cell line	Characteristics	Cell line	Characteristics	
HOS SaoS2	Elongated mitochondria	LM7 143B	Enlarged, rounded mitochondria; display mitochondrial swelling characteristics	Giang et al. (2013)
	Partial decrease of ΔΨm and mitochondrial integrity		Total decrease of ΔΨm and mitochondrial integrity	
	Downregulation of CytC and upregulation of Bcl-2, compared with non-cancerous hFOB cell line		Lower OCR and higher ECAR than hFOB and its parental counterparts	
			Unchanged basal rates of apoptosis	
			Upregulation of Bcl-2, compared with hFOB cell line	
MG63		MG63.3	Partial dependence to glucose, confirmed in glucose-deprived conditions and a total dependency to glutamine for growth	Ren et al. (2020)
**Differentiated OS model**		**Stem-cell OS model**		
MG63	Glucose-independent and addicted to glutamine	3AB-OS	Elongated mitochondrial network	(Palorini et al., 2014, p.)
	Higher mitochondrial activity		Reduce of mitochondrial respiration	
			Lower ΔΨm and number of mtDNA per cell content than MG-63	
			Higher basal of ROS	
			Glucose-dependent	

In contrast, metastatic LM7 and 143B cell line models contain many enlarged rounded mitochondria, showing mitochondrial swelling characteristics. These results were also confirmed in mouse 143B xenografts and diagnosed human OS biopsy specimens without any previous treatments. Therefore, metastatic LM7 and 143B cell lines showed significantly lower OCR and higher ECAR, compared to their non-cancerous and parental counterparts, indicating the presence of Warburg effect in metastatic OS cell lines. Furthermore, the ΔΨm and the mitochondrial membrane integrity were totally reduced, indicating mitochondrial depolarization, compared with these counterparts (Giang et al., 2013). As a consequence of these changes, the opening of mPTP and the release of apoptogenic factor—CytC were expected (Li et al., 1997) (Kalkavan and Green, 2018). Surprisingly, however, the protein expression of cytosolic CytC, caspase-3 was not significantly changed in 143B and LM7 cell lines. This was in line with the unchanged basal rates of apoptosis. Bcl-2 overexpression was also observed in LM7 and 143B, indicating the inhibition of mPTP opening mechanism and CytC release to induce apoptosis. Interestingly, by using an mPTP inhibitor named sanglifehrin A (SfA), the Warburg effect was significantly reversed in LM7 and 143B cell lines, accompanied by the increase of OCR and decrease of ECAR. As expected, the ΔΨm and integrity were consequently increased. These results indicate an interplay between mPTP, Warburg effect and mitochondrial dysfunction in OS (Giang et al., 2013).

Moreover, it has recently been shown that the cell energetic profile measured as the OXPHOS/glycolysis ratio might reflect the stemness of OS cells. Della Sala et al. (2022), compared the differentiated MG63 cell line with its OS subclone, the mesenchymal stem cell line 3AB-OS. This comparison showed that the stem cell like 3AB-OS cells display a more glycolytic profile. Interestingly, the reduced mitochondrial respiration of 3AB-OS cell line also correlates with a lower ΔΨm and mtDNA copy number, whereas MG-63 shows an elongated, and thus more functional mitochondrial network. The 3AB-OS cell line also produces higher basal levels of total ROS than MG63. Finally, by suppressing nutrient availability, two distinct energetic dependencies were observed, revealing that the 3AB-OS cell line is highly dependent on glycolysis while MG63 cells are addicted to glutamine (Palorini et al., 2014). These observations are similar to normal stem cells that often rely mainly on glycolysis and might be linked to the self-renewal capacity of 3AB-OS (Ito and Suda, 2014). To activate differentiation, stem cells need to reprogram metabolism towards OXPHOS. In line with this finding, high basal levels of ROS production in 3AB-OS enhance the glycolytic profile and inhibit the OXPHOS activity. With this mechanism they produce sufficient ATP for energy-consuming processes while maintaining stemness characteristics (Faubert et al., 2020) (Zhang et al., 2012) (Intlekofer and Finley, 2019). Contrary to 3AB-OS stem cells, differentiated MG63 cells are glucose-independent, but display glutamine addiction and higher mitochondrial activity (Palorini et al., 2014). Taken together, understanding of how OS stem cell metabolically reprogram could contribute to discover the new therapeutics directly targeting this chemoresistant sub-population in OS.

Amino acid and fatty acid metabolisms have also been described to be changed during OS oncogenesis. This includes 3-phosphoglycerate dehydrogenase (PHGDH) that has recently been identified as a potential target for OS treatment (Rathore et al., 2021). PHGDH is responsible for the conversion of the glycolytic intermediate 3-phosphoglycerate into 3-phosphohydroxypyruvate, an intermediate of *de novo* serine biosynthesis. Upregulation of PHGDH has been observed in many cancers, including melanoma, breast cancer, leukemia and colorectal cancer (Locasale et al., 2011) (Fan et al., 2015) (Polet et al., 2016) (Jia et al., 2016) (Zhao et al., 2020). This upregulation is thought to be linked to increased demands for folate and methionine cycle-related processes such as DNA synthesis, biomass production and epigenetic modifications (Rathore et al., 2020). Targeting the folate cycle is a mainstay of treatment in several cancers, including OS, with MTX being one of the oldest and still most widely used chemotherapeutics (Ghodke-Puranik et al., 2015). Given the role of MTX in inhibiting dihydrofolate reductase (DHFR), a component of the folate cycle, targeting PHGDH could support the use of MTX either to produce synergistic activity or to prevent current resistance issues in OS treatment, given that the molecules are non-cytotoxic. By analyzing 260 OS patient samples using both immunohistochemistry and RNA-sequencing analysis, Rathore et al. (2021) observed that patients expressing high PHGDH levels have worse outcomes and disease-free survival than low-PHGDH expressing patients. Moreover, a panel of OS cell lines was detected with three to ten times higher PHGDH protein expression, compared with precursor mesenchymal stem cell (hMSC). Those include MG63, HOS/MNNG, Saos2, U2OS, and NOS1. Interestingly, pharmacologically or shRNA targeting PHGDH showed decreased proliferation rates in OS cell lines, but no effects were shown in breast cancer cell lines with low PHGDH expression, thus confirming a potential therapeutic interest. Metabolic evaluation of PHGDH-inhibited OS cell lines showed a decrease of mitochondrial OCR and an increase of the glycolytic rate. Metabolomics analysis of those cells displayed a decrease of metabolites involved in the TCA cycle, suggesting that there might be a decreased glucose consumption to enter the TCA cycle for mitochondrial OXPHOS. In line with the glutamine addiction that we discussed above, the accumulation of glutamine and glutamate suggested that PHGDH-inhibited OS cell lines decrease glutamine consumption as a oxidative fuel source. To determine the alternative energy sources in PHGDH inhibitor (NCT-503)-treated OS cell lines, intracellular [U-^13^C] tracing analysis revealed the metabolic switch from glutamine utilization to fatty acid oxidation as a principal source of non-glucose-derived acetyl-coenzyme A (Acetyl-CoA), confirmed by the accumulation of unsaturated fatty acid by lipidomic analysis. Most strikingly, this was not sufficiently incorporated in the TCA cycle. The lipidomic analysis demonstrated that the increase of unsaturated fatty acids inside PHGDH-inhibited OS cell line was likely from dysfunctional mitochondria, rather than fatty acid biosynthesis. The exact mechanism, however, needs to be shown. In addition, inhibition of PHGDH also resulted in an increase of branched-chain amino acid (BCAA) metabolite levels (Rathore et al., 2021). Given the cell capacity to sense lipid, BCAA, methionine cycle, and nucleotide level changes, likely *via* GATOR complexes, mTORC1 and autophagic actor Atg4 were found to be activated to prevent mitochondria dysfunction and toxicity induced by pharmacological PHGDH inhibitor NCT-503 (Gu et al., 2017) (Kim et al., 2015). To counteract these compensatory effects, the mTORC inhibitor (rapamycin) was combined with NCT-503. In practice, however, this drug combination showed no difference in OS cell death, compared to monotherapy. Taking into account the metabolic compensation, observed with the accumulation of unsaturated fatty acid, while treated with the PHGDH inhibitor; perhexiline, the inhibitor of β-oxidation, and carnitine palmitoyltransferases 1/2 (CPT1/2) was combined with NCT-503 (Rathore et al., 2021). CPT1 and CPT2 are mitochondrial enzymes, responsible for the transport of acyl-carnitines into IMS to participate in fatty acid β-oxidation (Bonnefont et al., 2004). It was shown, that mono-treatment by perhexiline did not induce significant cell death while the combination of perhexiline with NCT-503 or of other PHGDH inhibitor, namely PKIMDL, caused significant cell death in OS cell line and also in U2OS xenograft models (Rathore et al., 2021). Despite the promising effects of PHGDH inhibitor in pre-clinical models and the discovery of new PHGDH inhibitors during this past decade, none of these inhibitors was demonstrated in clinical trials due to their high concentrations (µM range) and lack of sensitivity, thus leading to high toxicity. Therefore, the next challenging step of PHGDH inhibitor development is to identify new target action sites of that subsequently lower the concentration of those compounds and reduce the toxicity. In line with this concept, these results highlight the notion of metabolism flexibility in OS, thus constituting a potential Achilles Heel that can be leveraged to target metabolic vulnerabilities at different OS development stages. Despite a still very preliminary stage, this concept currently raises many therapeutic opportunities to precisely target metabolism (Wolpaw and Dang, 2018).

### 3.2 Multi-omics analysis to reveal metabolic reprogramming.

Recently, three human OS cell lines with the same genetic background, but at three different stages of OS development were compared by using metabolomic, proteomic, and lipidomic analysis, namely the benign HOS cell line, the malignant N-methyl-N′-nitro-N-nitrosoguanidine (MNNG) treated-HOS (MNNG/HOS) line and the metastatic HOS-derived 143B line (Table 2). Malignant MNNG/HOS and metastatic 143B cell lines exhibited the increase of glycolytic metabolites and the upregulation of glycolysis-involved enzymes than benign HOS cell lines with a non-significant difference between MNNG/HOS and 143B cell lines. Concerning the TCA cycle, MNNG/HOS displayed a higher level of TCA metabolites and TCA cycle-related enzymes than metastatic 143B cells. 143B cells also showed higher levels of both when compared to the benign HOS cells (MNNG/HOS >143B >HOS). The same classification was found in amino acid intermediate metabolites and enzymes responsible for amino acid metabolism. In the MNNG/HOS cell line, a significant 50% increase in lipid species was observed, in comparison to metastatic 143B cells. A pulse isotope approach was designed to trace the incorporation of ^13^C-glucose and ^13^C-glutamine. After feeding three cell lines with ^13^C-glucose, metastatic 143B cells showed significantly higher incorporation in glycolytic flux than their benign and malignant counterparts (Fritsche-Guenther et al., 2020). High levels of ^13^C-glutamine incorporation into the TCA cycle was also found in 143B, with predominant labeling of citric acid, suggesting a reductive carboxylation flux in the TCA cycle fuel Acetyl-CoA production (Martínez-Reyes and Chandel, 2020). Under glucose or glutamine deprivation, no significant change in cell viability was found in 143B, confirming the flexibility in using either glucose or glutamine for proliferation. In contrast the viability of HOS and MNNG/HOS was found to be reduced in glutamine-deprived conditions (Fritsche-Guenther et al., 2020).

**TABLE 2:** Metabolic reprogramming profiling by omics- analysis in OS.

Non-metastatic model		Metastatic model		References
Cell line	Characteristics	Cell line	Characteristics	
HOSMMNG/HOS	Lower expression of TCA cycle and amino acid -related enzymes and metabolites than malignant MNNG/HOS and 143B cell lines	143B	Higher expression of glycolytic metabolites and glycolysis-involved enzymes with a non-significant difference between MNNG/HOS and 143B cell lines	Fritsche-Guenther et al. (2020)
	Lower expression of lipid compounds than MNNG/HOS and 143B cell line		Lower expression of TCA cycle and amino acid -related enzymes and metabolites than malignant MNNG/HOS cell line but higher than HOS cell line	
	Reduced viability in glutamine-deprived conditions		Lower expression of lipid compounds than MNNG/HOS cell line but higher than HOS cell line	
			Metabolic flexibility in using either glucose or glutamine for its proliferation	
HOS	Lower expression of phosphatidylethanolamines, phosphatidylcholines, lysophosphatidylcholines, and ceramides than non-cancerous hFOB cell line	143B	Lower expression of phosphatidylethanolamines, phosphatidylcholines, lysophosphatidylcholines, and ceramides than non-cancerous hFOB cell line	Roy et al. (2019)
	Upregulation of cardiolipins		Higher expression of monoacylglycerol and diacylglycerol than hFOB and HOS cell lines	
			Lowest expression of lysophosphatidylcholine, lysophosphatidylethanolamine, phosphatidylglycerol, triacylglycerol, cholesteryl esters	
**3 weeks of LM8 inoculation in mice (starting point of metastasis)**		**4 weeks of LM8 inoculation in mice (lung metastasis)**		
Increases of glycolysis, TCA cycle-related metabolites, 2-HG, succinic acid, and most of amino acids, including serine and methionine		Decrease of carbohydrate and amino acid metabolism		Hua et al. (2011)
		Increase of fatty acid metabolism		

Further research on nutrient dependencies showed that the OS MG63-derived metastatic MG3.3 cell line shows a partial dependency on glucose and a total dependency to glutamine for growth. Upon treatment with a glutamine synthase (GLS) inhibitor, only a partial reduction of cell growth was observed, suggesting the capacity of these cells to switch the primary nutrient source. Increased, growth inhibition was observed *in vitro* as well as in an orthotopic xenograft model, by combining the GLS inhibitor with metformin, a mitochondrial Complex I (CI) inhibitor (Ren et al., 2020). The mechanisms of action of metformin in OS treatment will be discussed below in more detail.

In addition to the analysis of tumor tissue, Hua et al. (2011) performed a metabonomic study of serum in mice carrying subcutaneous tumors of murine LM8 OS cell lines. In this model all mice developed lung metastasis after 4 weeks of LM8 inoculation, their serum metabolic profiles revealed dynamic changes associated with the development of OS metastasis. In the third week, considered as the starting point of metastasis, many glycolysis and TCA cycle-related metabolites were up-regulated in serum which is consistent with the Warburg effect previously observed in the comparison of Saos2, HOS cell lines and its metastatic subtypes LM7, 143B (Hua et al., 2011) (Giang et al., 2013). Among dysregulated metabolites, the level of 2-hydroxyglutaric acid (2-HG) and succinic acid were significantly upregulated in the third week. Moreover, most of the amino acids were upregulated, including serine and methionine which are correlated with studies of Rathore et al. (2021), thus constructing a specific metabolic signature of OS. At week 4 when the lung metastasis is fully developed, metabolic reprogramming was observed with the decrease of carbohydrate and amino acid metabolism whereas fatty acid metabolism was increased, indicating a role of lipid metabolism in lung metastasis in this model (Hua et al., 2011).

2-HG is considered as an oncometabolite and strongly contributes to tumorigenesis in many cancers (Parsons et al., 2008) (Mardis et al., 2009) (Shelar et al., 2018). Mutated forms of isocitrate dehydrogenase 1/2 (IDHmt1/2) that catalyze the conversion from α-ketoglutarate to the D enantiomer of 2-HG (D-2-HG) were famously identified as potential targets with the development of the first-in-class metabolic inhibitors (Parsons et al., 2008) (Dang et al., 2009). In 2017 and 2018, these metabolic inhibitors, namely Enasidenib and Ivosidenib, have been approved by the US Food and Drug Administration (Kim, 2017) (Dhillon, 2018). Currently, Ivosidenib is explored in the Phase II clinical trial in advanced solid pediatric tumors, for pediatric patient containing *IDH1* genetic alterations, including OS (NCT04195555) (National Cancer Institute (NCI), 2022) and Enasidenib in *IDH2*-mutated malignancies in the European AcSé-ESMART trial (NCT02813135) (Gustave Roussy, Cancer Campus, Grand Paris, 2021) (Gatz et al., 2019). Despite many different studies reporting that no mutations of neither *IDH1* nor *IDH2* were detected in OS patient samples, Liu et al. (2013) nonetheless demonstrated that IDH2-R172S was still detected in 25% of OS patients by using their anti-mutated monoclonal antibody MsMab-1 (Amary et al., 2011). Taking into account that for acute myeloid leukemia, serum 2-HG levels have been discovered as a diagnostic and prognostic tool to predict IDHmt1/2 and also clinical outcome, the detection of IDHmt1/2 needs to be re-examined by different methods in OS (DiNardo et al., 2013). Of note, only D-2-HG is increased in IDHmt1/2, the metabolic difference that Hua and colleagues reported, could be the total amount of D-2-HG and L-2-HG. Therefore, the ratio of these two enantiomers or the separated quantification of D-2-HG becomes critical for specific clinical test development to avoid false-positive results in the future (Struys, 2013).

A detailed lipidomic analysis of the non-cancerous hFOB, non-metastatic HOS, and metastatic 143B was performed by Roy et al. (2019). Many chief lipid classes were found to be differentially regulated. Briefly, phosphatidylethanolamines, phosphatidylcholines, lysophosphatidylcholines, and ceramides displayed the highest expression, while monoacylglycerol and diacylglycerol displayed the lowest expression in hFOB, compared with HOS and 143B cell lines. When evaluating the functional consequences, they could show that inhibiting phospholipase C (PLC), whicht catalyzes the hydrolysis of phosphatidylinositol 4,5-bisphosphate (PIP2) into diacylglycerol and IP3, reduces 143B cell viability and migration. Moreover, several interesting observations were obtained that could be further investigated in the future: 1) cardiolipins are upregulated in the HOS cell line, while there was no significant difference between hFOB and 143B, and 2) lysophosphatidylcholine, lysophosphatidylethanolamine, phosphatidylglycerol, triacylglycerol, cholesteryl esters display the lowest poolsize in 143B cells. Upon more detailed examination of cholesterol species levels (cholesterol, free cholesterol, and cholesteryl ester), no significant changes between cell lines were observed (Roy et al., 2019). The only significant change was the decrease of esterified cholesterol in 143B, compared with hFOB and HOS. The authors hypothesized that this decrease was due to the lack of adipocytes in their cellular model and the result could not reflect the *in vivo* condition. In conclusion, further investigation of lipidomic profiling into *in vivo* primary and metastatic OS models could confirm their observations and enable targeting lipid synthesis in OS treatment (Roy et al., 2019).

## 4 Two examples of on-going clinical trials of metabolic inhibitors for osteosarcoma treatment

In this review, we have searched in ClinicalTrials.gov with “Osteosarcoma” for condition or disease, “Metabolism” for other terms and selected “Recruiting” and “Active, not recruiting” for recruitment status. Thus, five studies were found to be relevant with these terms. Among these five studies, there are only Metformin and combination of ADI-PEG20 with Gemcitabine and Docetaxel that showed the complete matched criteria. As consequence, we finally decided to decipher Metformin and ADI-PEG20 because of their active clinical status to summarize the real-time advances in using metabolic inhibitors in OS treatment.

### 4.1 Metformin

Metformin has originally been developed as a biguanide antidiabetic agent to treat type II diabetes mellitus (Rena et al., 2017). In recent years, metformin has been evaluated in a wide variety of cancer models for its effect on cell viability and growth. Despite the very high concentration of metformin was reported to have effect on tumor growth *in vitro*, it is still one of the most prescribed drugs worldwide for this indication. Metformin is transported into the cell *via* organic cation transporter 3 (OCT-3) and OCT-1 (Jonker and Schinkel, 2004). The first known target of Metformin is complex I of mitochondrial electron transport chain (mtETC), exhibiting the antitumor effects through the decrease of respiration chain activity and oxygen consumption rate (El-Mir et al., 2000). Moreover, it has been shown that Metformin treatment influences multiple signaling pathways involved in cellular proliferation and survival such as AMPK signaling pathway and mTORC1 pathway. By inhibiting mitochondrial complex I, metformin treatment increases the ADP: ATP and AMP:ATP ratios, leading to the activation of AMPK signaling pathway. The AMPK pathway activation results in suppression of hepatic gluconeogenesis, inhibition of lipogenesis by reducing the conversion of acetyl-CoA to malonyl-coA (Musi et al., 2002) (Foretz et al., 2014) (Whitburn et al., 2017). According to the literature, the anti-cancer effects of metformin were also described by the APMK-induced inhibition of mTOR activity, that, subsequently, inhibits the protein synthesis (Howell et al., 2017). Accumulated evidences suggested that metformin could target the HK-VDAC1 interaction by allosterically inhibiting HK enzymatic activity, leading to detach HK from mitochondrial VDAC1 and promotes apoptotic cell death (Marini et al., 2013) (Abu-Hamad et al., 2008) (Shoshan-Barmatz et al., 2021).

Several clinical studies in different cancer types have been using metformin as mono-treatment, or combination with other molecules to improve therapeutic response (J. Li et al., 2020b). NCT01101438 is an active phase III clinical trial that showed the improvement of patient’ progression-free survival while treated with Metformin for more than 3,000 breast cancer patients (Canadian Cancer Trials Group, 2022). Several on-going clinical trials are promisingly conducted by combinating Metformin with targeted therapy such as BRAF tyrosine-kinase inhibitors (TKI) for melanoma (NCT01638676) (NCT02143050) (Chesney, 2021a) (Chesney, 2021b). Temsirolimus (mTOR inhibitor) for advanced cancers patients that are refractory to standard therapies (NCT01529593) (M.D. Anderson Cancer Center, 2022a), Sapanisertib (mTOR1/2 inhibitors) for metastatic, recurrent and refractory cancers (NCT03017833) (M.D. Anderson Cancer Center, 2022b). Combining Metformin with standard chemotherapy has also shown the synergetic effects and been conducted in several on-going clinical trials for breast cancer (NCT03238495) (Khan, 2021) and prostate cancer (NCT02640534) (Swiss Group for Clinical Cancer Research, 2022). Of note, Metformin has no significant side effects; however, the overdose of Metformin can cause the symptoms of lactic acidosis that likely occur in patients with pre-existing medical conditions and those older than 80 years old (Nasri and Rafieian-Kopaei, 2014). In the population-based study of type II diabetes patients, nearly 25% of patients have contraindications to Metformin treatment, but rarely results in discontinuation of therapy (Emslie-Smith et al., 2001, p.). However, the benefit-risk balance of metformin treatment in cancer therapy still requires to be further investigated.

In OS cell lines, metformin exhibited a dose- and time-dependent inhibition of cell cycle, tumor proliferation, and invasion *in vitro* and in xenograft models, as shown by three different research groups (Ko et al., 2016) (B. Li et al., 2020a) (Li et al., 2018). In these studies, high concentration (mM range, far higher than doses used in patients) of metformin could trigger cell cycle arrest by G2/M accumulation, which was confirmed by the upregulation of cell cycle-related protein Cyclin D1. Metformin-induced apoptosis was also found as indicated by loss of ΔΨm, shrinkage of the cells, fragmentation of nuclei, and the cleavage of caspase-3 and PARP. Interestingly, metformin-treated OS cell lines also manifested higher levels of autophagy-related actors such as LC3B-II, p62 and Beclin-1. To determine the crosstalk between apoptosis and autophagy they co-administered an autophagy inhibitor with metformin. This further improved the anti-tumor effect of metformin on OS tumor growth, indicating an anti-apoptotic effect of autophagy in OS treatment. Similarly, in other model systems, it has been proven that mitochondrial ROS play a crucial role in the activation of apoptosis and autophagy *via* the ROS/JNK/c-Jun pathway. In analogy, mitochondrial ROS production was increased in metformin-treated OS cell lines (B. Li et al., 2020a). According to these results, metformin activates JNK/c-Jun pathway-mediated autophagy and apoptosis by stimulating the phosphorylation of JNK and c-Jun (Redza-Dutordoir and Averill-Bates, 2016) (Wang et al., 2016).

Metastasis development is strictly linked to the EMT mechanisms and the loss of cellular adhesion that facilitates cell migration. On immunohistochemistry, this is characterized by decreased expression of epithelial markers (E-cadherin and ZO-1) and increased expression of mesenchymal markers (N-cadherin and vimentin) (Greenburg and Hay, 1982) (Stoker and Perryman, 1985). Beyond the known role of metformin on mitochondrial CI, metformin-treated OS cell lines showed an increase of epithelial marker E-cadherin and a decrease of the mesenchymal marker vimentin, suggesting that metformin could negatively regulate the EMT mechanism (Ren et al., 2020) (Li et al., 2018). A proposition of explanation of the mechanism of action of high doses of metformin would be that phosphatase and tensin (PTEN) tumor suppressor activity is upregulated and thus, phosphorylated Akt (p-Akt) were found to be decreased (Li et al., 2018). It is known that PTEN/PI3K/Akt pathway hyperactivation is crucial in many cancers, including OS and that the genomic deletion of *PTEN* gene is frequently observed in OS cell lines (Freeman et al., 2008) (Zheng et al., 2020) (Nielsen-Preiss et al., 2003). In further detail, immunohistochemical stainings of PTEN of nearly 100 OS patients were analyzed and divided into two groups: 1) chemotherapy naive biopsies (BXs) and 2) tumor resections after neoadjuvant chemotherapy (RXs). BXs significantly displayed a positive staining for two thirds of samples whereas RXs after receiving chemotherapy, showed the decrease of PTEN marker. Moreover, patients with low PTEN expression are associated with poorer prognosis in both BXs and RXs (Robl et al., 2015). As a well-defined pathway, a product of PI3K - phosphatidylinositol-3-phosphates (PIP-3) level is maintained low by PTEN activity. Consequently, the loss of PTEN increases PIP-3 level, and the accumulation of PIP-3 recruits and hyper-activates several proteins containing pleckstrin homology (PH) domain, including Akt (Di Cristofano and Pandolfi, 2000). Indeed, increased proliferation, migration and invasive rates were found in PTEN-knocked-down Metformin-treated OS cell lines, compared with only Metformin-treated OS cell lines, thus indicating that the anti-tumor effect of metformin was suppressed by prior PTEN knock-down using siRNA (Li et al., 2018).

**FIGURE 6:**
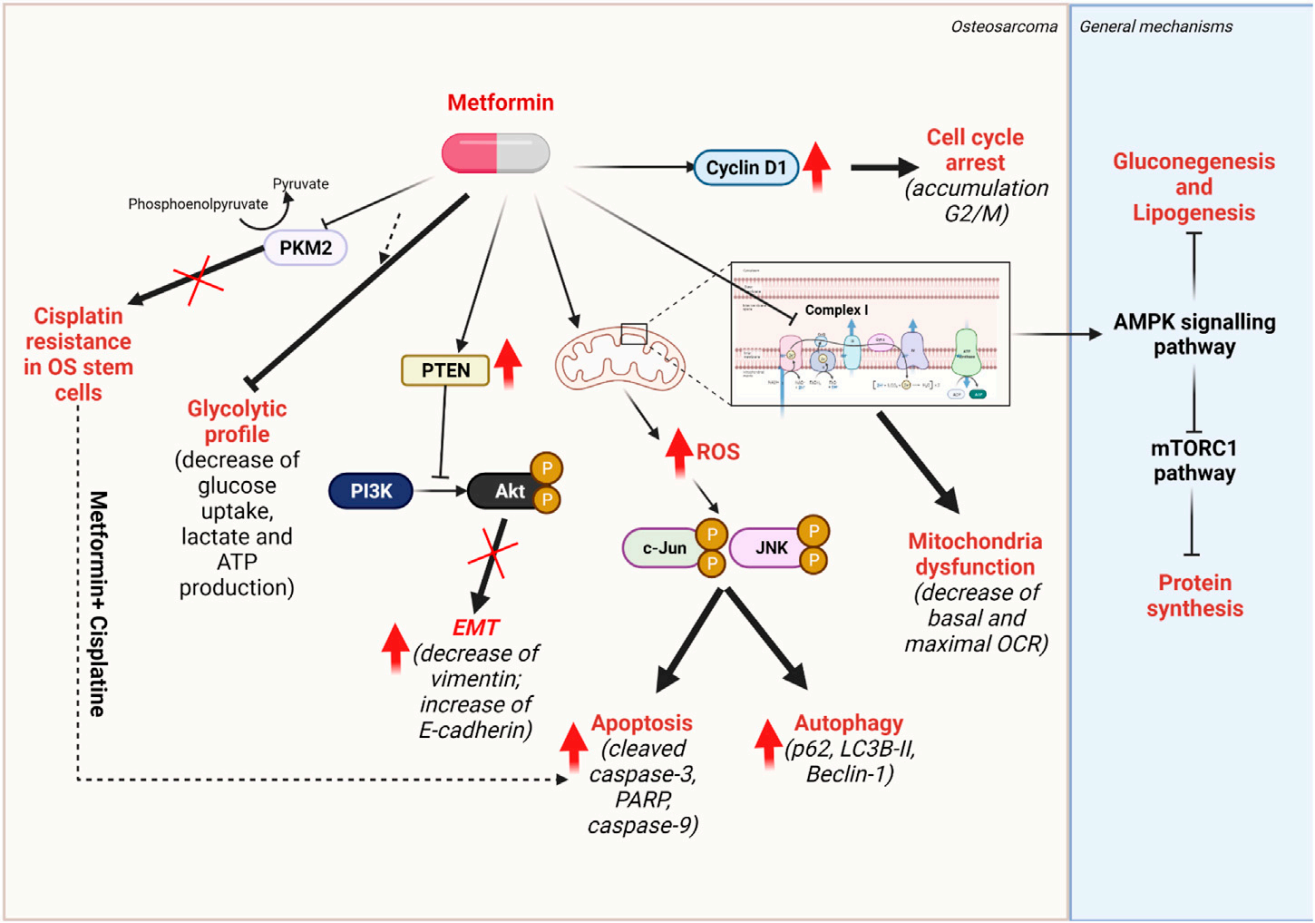
Mechanisms of action of metformin. Metformin targets mitochondrial complex I of respiratory chain, thus induces the activation of AMPK signalling pathway, resulting in the inhibition of glucogenesis and lipogenesis. The overactivation of AMPK pathway also inhibits the mTORC1 pathways, thus, protein synthesis.

Moreover, metformin can sensitize OS stem cells to CIS by inhibiting the pyruvate kinase isoenzyme M2 (PKM2) which is consistent with many different studies in other cancers (Shang et al., 2017, p. 2) (Chen et al., 2015) (M. Liu et al., 2019b, p. 2). Distinct mechanistic investigations were conducted in different cancer models. In gastric cancer, metformin decreases PKM2 protein expression *via* HIF1α inhibition, while in renal cancer cell, metformin promotes the activation of AMPK under glucose deprivation, thus resulting in PKM2 recruitement to form AMPK/PKM2/β-Catenin complex that subsequently stimulates the transcription of *c-Myc* and *CCND1* genes (Chen et al., 2015) (M. Liu et al., 2019b, p. 2). By sorting OS cell lines (HOS, Saos2 and MG-63) with CD133 markers, Shang et al. (2017) were able to separate CD133-positive OS stem cells and CD133-negative OS non-stem cells. In this study, it was observed that CD133-negative OS non-stem cells responded better to CIS than CD133-positive OS stem cells. In OS stem cells, the expression of PKM2 is significantly higher than in OS non-stem cells. To confirm the role of PKM2 in CIS resistance, PKM2-knocked-down OS stem cells showed significantly higher sensitivity to CIS treatment. In line with PKM2-involved CIS resistance, metformin treatment appears to sensitize OS stem cells to CIS by inhibiting PKM2 in both *in vitro* and *in vivo* models. Regarding metabolic impacts, metformin was able to lower glucose flux, as indicated by reduced glucose uptake, lactate production, and ATP production. This might partially explain the effect of metformin on tumor proliferation. Combining metformin and CIS resulted in higher expression of cleaved caspase-9 and cleaved caspase-3, effectors of apoptosis (Shang et al., 2017, p. 2) (Figure 6).

Based on different preclinical studies of metformin, a phase-II single-arm clinical trial of metformin as 3 years maintenance treatment for OS patients is currently recruiting (NCT04758000). The study is divided into two groups, the first group of poor responders for post neoadjuvant chemotherapy and the second group of patients (including OS patients and Ewing sarcoma patients) in complete remission after the first relapse to clinically evaluate the event-free survival and toxicity of metformin (Longhi, 2021).

Metformin resensitizes CIS-resistant OS stem cells to CIS and promotes apoptosis by increasing levels of cleaved caspase-3 and caspase-9; it induces reduced glycolytic flux as indicated by decreased glucose uptake, lactate, and ATP production; it prevents the EMT-mediated metastasis by the loss of mesenchymal marker vimentin and the gain of epithelial marker E-cadherin *via* the increase of PTEN, resulting in inhibiting cell survival PI3K/Akt pathway. Metformin targets the complex I of mtETC and alters mitochondrial activity. Thus, an over-production of ROS was found to promote both apoptosis and protective autophagy *via* the JNK/c-Jun pathway. Cell cycle arrest, characterized by the cell accumulation in the G2/M phase, was found in metformin-treated OS cell lines by elevating Cyclin D1 protein expression.

### 4.2 ADI-PEG20 combined with Docetaxel and Gemcitabine

**FIGURE 7:**
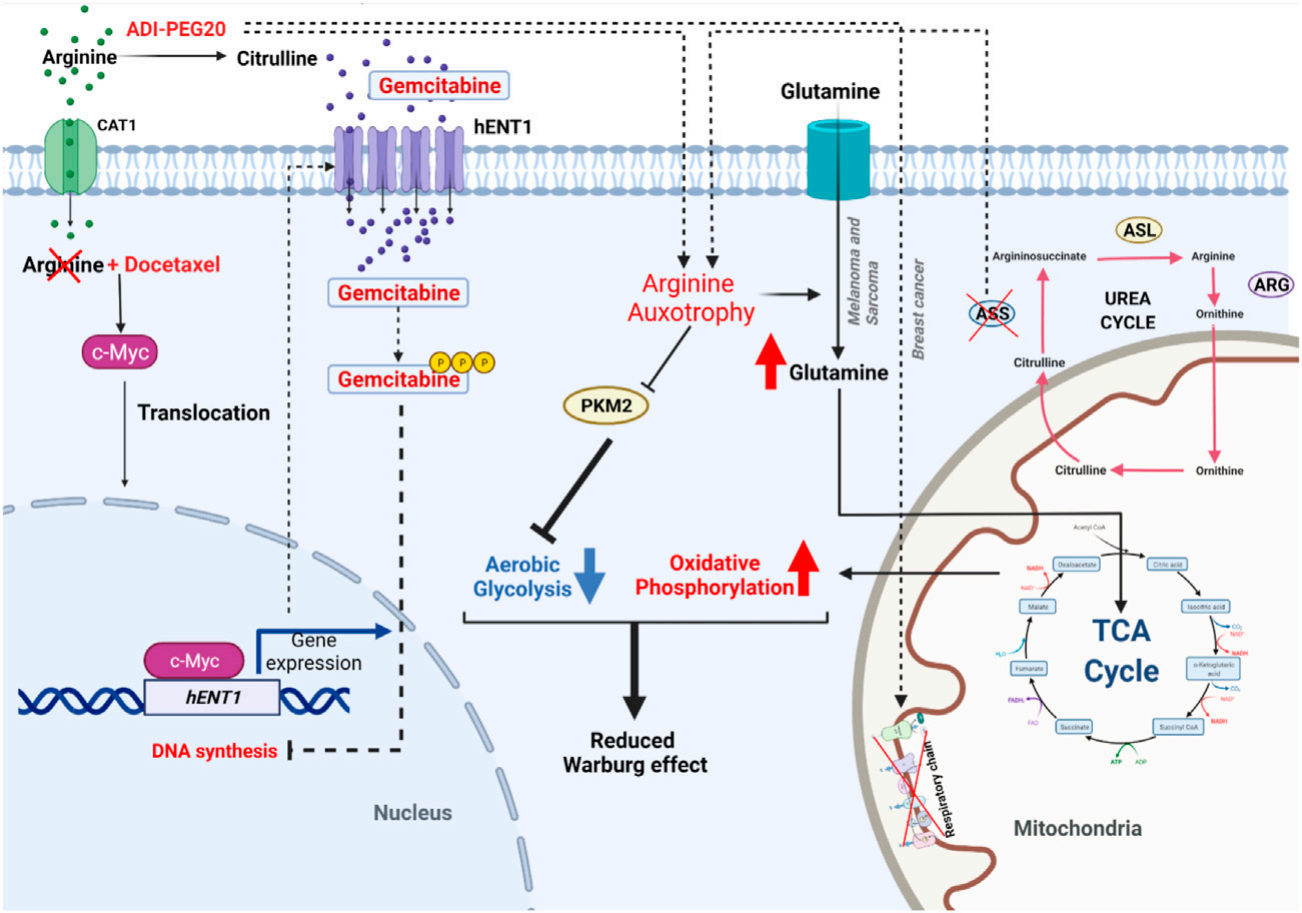
Mechanisms of action of ADI-PEG20 combined with Docetaxel and Gemcitabine in Ongoing Clinical for *ASS1*-deficient cancer (adapted from Prudner et al., 2019). Shown on the left are the synergic molecular mechanism of ADI-PEG20, Docetaxel, and Gemcitabine in *ASS1*-deficient OS. Together with Docetaxel, the arginine starvation induced by ADI-PEG20 induces c-Myc translocation. c-Myc acts as a transcription factor that stimulates the expression of the *hENT1* gene which is important for Gemcitabine uptake to inhibit DNA synthesis in OS. Shown on the right are the metabolic impacts in arginine auxotrophic conditions, where the loss of arginine inhibits the activity of glycolysis-mediated enzyme PKM2, resulting in decrease of aerobic glycolysis. Moreover, in *ASS1*-deficient breast cancer, ADI-PEG20 treatment inhibits the mitochondrial activities, resulting in the inhibition of mitochondrial respiratory complexes. While in melanoma and sarcoma, arginine starvation causes the increase of extracellular glutamine uptake with higher expression of glutaminolysis-related actors, resulting in the metabolic reprogramming for the consumption of glutamine as a fuel source *via* TCA cycle, and OXPHOS. This metabolic profile represents the reduced Warburg effect.

Due to the deficiency of cellular arginine synthesis, many cancer cells depend on the uptake of exogenous arginine when citrulline or ornitine uptake is not possible (Cheng et al., 2018). Whereas the full urea cycle incorporates carbamoyl phosphate that stems from the assimilation of ammonia with bicarbonate; also, partial cycles exist starting with citrulline, taken up from circulation. Citrulline then enters the urea cycle and generates arginine *via* two reactions, catalyzed by argininosuccinate (ASS) for the conversion from citrulline + aspartate to argininosuccinate and argininosuccinase (ASL) for the conversion from argininosuccinate to arginine + fumarate (Figure 7).

Focusing on the rate-limiting enzyme ASS, the isoform ASS1 is commonly lost in high-grade sarcoma, its role being crucial for arginine production. Therefore, the loss of ASS1 is considered a metabolic vulnerability because, growth of *ASS1*-deficient cancer cells under arginine depletion is strongly impaired (Bean et al., 2016). To investigate this treatment strategy, starvation of exogenous arginine was induced by using arginine deiminase (ADI-PEG20). This enzyme degrades extracellular arginine to citrulline, and results in loss of exogenous arginine uptake *via* CAT1 transporter (Closs et al., 2004). ADI-PEG20 has been investigated in many different cancer types (Kobayashi et al., 2010) (Huang et al., 2013) (Bowles et al., 2008).

In ASS1- deficient breast cancer, arginine starvation by ADI-PEG 20 displayed the dysregulated mitochondrial bioenergetics by decreasing 50% of total ATP levels and inhibiting oxygen consumption rate in MDA-MB-231 cells. Transcriptomic analysis comparison between treated and un-treated MDA-MB-231 cell lines showed several mitochondrial candidates such as members of complex I, complex II, complex III, complex IV and complex V of mtETC, thus confirming the effect of ADI-PEG20 on mitochondrial respiratory function. Moreover, ADI-PEG20 treatment results in ROS over-production, decrease of mitochondrial membrane potential, mitochondrial fragmentation in ASS1-deficient MDA-MB-231 cells. Furthermore, in this breast cancer model, Qiu et al. (2014) combined ADI-PEG20 with autophagy inducer (rapamycin) or autophagic flux inhibitor (chloroquine) to observe the requirement of autophagy in ADI-PEG-induced cell death. Interestingly, chloroquine, not rapamycin was able to decrease ADI-PEG20-induced cell death. As consequence, they proposed that prolonged ADI-PEG20 treatment induces cytotoxic autophagy-dependent cell death. In contrast, the biological differences between melanoma and sarcoma versus breast cancer were observed in other study conducted by Kremer et al. (2017) in leiomyosarcoma and melanoma cell lines. Upon ADI-PEG20 treatment, the glucose uptake was reduced with the lower expression of PKM2, whereas the OXPHOS activity was increased that may explain. In line with this study, intracellular [U-^13^C] tracing analysis revealed that intermediate metabolite levels of pentose phosphate pathway (6-phosphogluconolactone), citrate and lactate were significantly decreased, indicating the inhibition of the Warburg effect and cellular glucose dependence in *ASS1*-deficient cell lines. Moreover, arginine starvation also caused the increase of extracellular glutamine uptake with higher expression of glutaminolysis-related actors such as GLS and glutamate dehydrogenase. This process determines the metabolic switch from aerobic glycolysis to glutamine metabolism to fuel OXPHOS activity through the TCA cycle in ADI-PEG20 treated cell lines. This metabolic vulnerability was then explored using the combination of ADI-PEG20 and GLS inhibitor bis-2-(5-phenylacetamido-1,3,4-thiadiazol-2-yl)ethyl sulfide (BPTES), resulting in better reduction in a broad range of ASS1-deficient cell lines, including OS cell line.

However, the only concern to be taken into account is that ADI-PEG20 can stimulate the re-expression of *ASS1* gene due to metabolic adaptation, resulting in the resistance mechanism of *ASS1*-deficient cancer cells (Shen et al., 2003) (Feun and Savaraj, 2006). The study conducted by Prudner et al. (2019) showed the promising combination of arginine deiminase (ADI-PEG20), Gemcitabine, and Docetaxel on a panel of *ASS1*-low to -null expressing cancers, both *in vitro* and *in vivo.* This study cohort included not only OS, but a larger group of sarcomas including fibrosarcoma, uterine spindle cell sarcoma, leiomyosarcoma, melanoma, Ewing sarcoma and rhabdomyosarcoma. Of note, Gemcitabine is well known as a nucleoside analog that incorporates into the DNA and induces cell deaths, used for many cancer treatments (de Sousa Cavalcante and Monteiro, 2014) (Toschi et al., 2005). Docetaxel is a microtubule-binding agent, causing cell-cycle arrest and apoptosis (Montero et al., 2005). The combination of Gemcitabine and Docetaxel is used as a second-line therapy for advanced soft-tissue sarcoma (Maki et al., 2007, p. 002) (Seddon et al., 2017). Gemcitabine resistance is associated to the dysregulation of the Gemcitabine metabolism-related proteins, including the decrease of Human Equilibrative Nucleoside Transporter 1 (hENT1)—the main plasma membrane transporter of Gemcitabine; decrease of deoxycytidine kinase (dCK)—a kinase responsible for the phosphorylation of Gemcitabine and increase of ribonucleotide reductase subunit M1 and 2 (RRM1/2) that can convert active Gemcitabine to its inactive form (Jia and Xie, 2015). Interestingly, in *ASS1*-deficient pancreatic cell lines, ADI-PEG20 can inhibit RRM2 while this effect was not observed in other *ASS1*-expressing cell lines. Of note, *RRM2* gene is negatively regulated by transcription factor E2F-1. Therefore, ADI-PEG20 increases E2F-1 and inhibits *RRM2* transcription. Thus, the combination of ADI-PEG20 and Gemcitabine exhibited an increased growth reduction, leading to tumor regression both *in vitro* and *in vivo* (Daylami et al., 2014). Other studies showed that the arginine starvation by ADI-PEG20 combined with the cMyc stabilization by Docetaxel increases expression of hENT1. The combination of these three drugs promotes the entry of Gemcitabine in cancer cells and results in higher cell death rate (Figure 7). Taking into account the re-expression of *ASS1* as a resistant mechanism, the research group showed that the long-term treatment with ADI-PEG20 remains effective (Prudner et al., 2019).

Based on these findings, a phase II clinical trial is now active, named “ADI-PEG 20” for combination with Docetaxel and Gemcitabine for the treatment of soft tissue sarcoma as the first cohort and OS, Ewing’s Sarcoma, Small Cell Lung Cancer as the second cohort for progression/relapse disease after failure of standard treatment (NCT03449901) (Washington University School of Medicine, 2021) (Figure 7). Of note, in the previous clinical evaluation of ADI-PEG20 in advanced hepatocellular carcinoma, the most frequent adverse event is fatigue and there is no statistical difference in term of adverse event between ADI-PEG20 treated patients versus placebo cohort (Abou-Alfa et al., 2018, p. 0).

## 5 Conclusion

Metabolic reprogramming is considered a central hallmark in cancer. It allows not only utilization of various energy sources depending on their availability in the tumor microenvironment, but most importantly fuel biomass synthesis. In line with the increased attention to this field across disciplines, we reflect in this summary on the current knowledge of cell death and metabolic reprogramming at different OS stages.

In this review, we discuss how mitochondria-related RCD and metabolism directly impact the proliferation of benign, malignant, and metastatic OS *in vitro* and *in vivo* models. We summarize the evidence that supports the interconnectivity of different mitochondria-related RCD pathways, with a focus on fundamental notions of apoptosis, ferroptosis, necroptosis, and autophagy and their involvement in cell fate, progression, and metastasis. The development of RCD inducers has recently emphasized the importance of a balance between apoptosis and autophagy, which is crucial for cell fate and drug efficacy. Autophagy also plays an essential role in the induction of ferroptosis and necroptosis by regulating metabolic pathways and ROS generation. Furthermore, necroptosis and extrinsic apoptosis share many common actors within the signal transduction pathways. Therefore, the crosstalk between apoptosis, ferroptosis, necroptosis, pyroptosis and autophagy creates an orchestra of RCD, emphasizing many new targets that could become useful suggestions for the development of OS therapeutic strategies in the future.

Many RCD and metabolism inducers or inhibitors are actively under investigation and have recently opened new areas for potential mono- or combinatorial OS therapies. However, at this stage undoubtedly further studies are needed to build fundamental knowledge of mitochondria-related RCDs and metabolic adaptation for novel OS treatments. Because of the high incidence of OS metastasis, relapse, and drug resistance, novel pharmacological approaches are urgently needed in this patient category. Currently, metformin and the combination of ADI-PEG20, Docetaxel, and Gemcitabine are in active clinical studies for OS treatment. This will thus provide a first benchmark of translational relevance of these findings and hopefully open the door to further novel treatment strategies for OS.

The question of how OS cells can precisely modulate cell death machineries and metabolism remains to be fully elucidated. Notably, comparing OS mitochondrial metabolism with that of other cancer types remains a future challenge and could be instrumental to develop novel OS therapeutic approaches in the near future.
